# Necroptosis: A Pathogenic Negotiator in Human Diseases

**DOI:** 10.3390/ijms232112714

**Published:** 2022-10-22

**Authors:** Hitesh Singh Chaouhan, Ch Vinod, Nikita Mahapatra, Shao-Hua Yu, I-Kuan Wang, Kuen-Bao Chen, Tung-Min Yu, Chi-Yuan Li

**Affiliations:** 1 Graduate Institute of Biomedical Sciences, China Medical University, Taichung 40402, Taiwan; 2 Department of Biological Sciences, School of Applied Sciences, KIIT University, Bhubaneshwar 751024, India; 3 Department of Emergency Medicine, China Medical University Hospital, Taichung 40402, Taiwan; 4 School of Medicine, China Medical University, Taichung 40402, Taiwan; 5 Department of Internal Medicine, China Medical University Hospital, Taichung 40402, Taiwan; 6 Department of Anesthesiology, China Medical University Hospital, Taichung 40402, Taiwan; 7 Division of Nephrology, Department of Internal Medicine, Taichung Veterans General Hospital, Taichung 40402, Taiwan

**Keywords:** necroptosis, RIPK1, RIPK3, MLKL, apoptosis, human diseases

## Abstract

Over the past few decades, mechanisms of programmed cell death have attracted the scientific community because they are involved in diverse human diseases. Initially, apoptosis was considered as a crucial mechanistic pathway for programmed cell death; recently, an alternative regulated mode of cell death was identified, mimicking the features of both apoptosis and necrosis. Several lines of evidence have revealed that dysregulation of necroptosis leads to pathological diseases such as cancer, cardiovascular, lung, renal, hepatic, neurodegenerative, and inflammatory diseases. Regulated forms of necrosis are executed by death receptor ligands through the activation of receptor-interacting protein kinase (RIPK)-1/3 and mixed-lineage kinase domain-like (MLKL), resulting in the formation of a necrosome complex. Many papers based on genetic and pharmacological studies have shown that RIPKs and MLKL are the key regulatory effectors during the progression of multiple pathological diseases. This review focused on illuminating the mechanisms underlying necroptosis, the functions of necroptosis-associated proteins, and their influences on disease progression. We also discuss numerous natural and chemical compounds and novel targeted therapies that elicit beneficial roles of necroptotic cell death in malignant cells to bypass apoptosis and drug resistance and to provide suggestions for further research in this field.

## 1. Introduction

Cell death plays an essential role in tissue homeostasis and embryonic development by eliminating unwanted cells, such as damaged cells [1]. Based on morphological features and molecular machinery, cell demise is attained through apoptosis, autophagy, necrosis, and mitotic catastrophe [2]. Apoptosis and necrosis represent the two most distinctive and best-understood cell death modalities. Apoptosis, also called programmed cell death (PCD), is a tightly regulated form of cell death, which is mediated through multiple enzymes leading to non-lytic cell death, characterized by specific morphological features such as cell shrinkage, apoptotic body formation, membrane blebbing, DNA fragmentation, and chromatin condensation [3]. Necrosis is a deregulated form of cell death induced via different external stressors such as infection, toxins, heat, and ischemia [4], which is characterized by an early loss of cell membrane integrity, swelling of sub-cellular organelles, and spillage of intracellular contents into extracellular space [5]. Decades ago, an alternative regulated mode of cell death was reported, where the traditional PCD pathway was found to be inhibited, and the mechanisms were functionally similar to apoptosis while morphologically similar to necrosis, hence known as necroptosis [6] (Table 1). Necroptosis involves formation of a necrosome, a complex consisting of RIPK1, RIPK3, and MLKL pseudokinases. It is stimulated by diverse factors such as the tumor necrosis factor (TNF) superfamily (TNFR1, Fas/CD95, and TRAIL-R), Toll-like receptors (TLR3 and TLR4), interferon-γ (IFN-γ), lipopolysaccharides (LPS), pathogen infection and various drugs [7,8]. Here, RIPK1 acts as a key regulator in necroptosis-mediated signaling, which is inhibited by necrostatin-1 (Nec-1), resulting in the occlusion of necroptosis [9,10]. Usually, necroptosis is mediated by death receptor signaling after the interaction of death-signaling molecules (TNFα and FAS) with their corresponding membrane receptors. Activated RIPK1 interacts with RIPK3 resulting in auto-phosphorylation of RIPK3, eventually culminating with the formation of the RIPK1-RIPK3-MLKL complex. RIPK3 further activates MLKL via phosphorylation, leading to the oligomerization of MLKL, which further translocates to the inner membrane of the plasma membrane and disturbs the integrity of the cell [11,12]. Furthermore, small amounts of evidence have demonstrated that ablation of caspase-8 leads to shifting of the apoptotic pathway toward the necroptotic mode of cell death and is considered as the decisive step for necroptosis [10,13,14]. In normal conditions, caspase-8 induces the apoptotic pathway by blocking necroptosis through cleaving RIPK1, RIPK3, and cylindromatosis (CYLD) [15]. During death receptor-induced necroptosis signaling, cIAPs (cellular inhibitor of apoptosis proteins) activate NF-κβ and MAPKs signaling by polyubiquitination of RIPK1, as a result of which RIPK1 cannot bind with caspase-8 and FADD, leading to impaired caspases activation, and thus inhibiting the initiation of apoptosis [16,17]. Inhibition of cIAPs or their signaling firmly shifts the pro-survival process into a pro-death process [18]. A study led by Feng et al. [19] demonstrated that RIPK1 and RIPK3 also play roles in caspase-independent cell death. Several studies showed that RIPK3 and MLKL disturb the integrity of the cell and play pivotal roles in necroptotic cell death [20,21], suggesting that there are two basic conditions for the occurrence of necroptosis: (1) cells must possess RIPK3 and MLKL and (2) inactivation of caspase-8 molecules. However, one of the in vivo studies reported conflicting results associated with necroptosis induction after inhibition of caspase-8 [22]. 

Furthermore, classical necroptosis is also involved in the induction of inflammatory responses driven by the excess secretion of inflammatory cytokines and the release of damage-associated molecular patterns (DAMPs) from the disintegrated cellular membrane, which is closely associated with different inflammatory diseases such as bowel disease, Crohn’s disease, psoriasis, and rheumatoid arthritis [30,31]. Moreover, necroptosis is also involved in many pathological conditions such as acute and chronic pulmonary diseases, renal, hepatic, cardiovascular and neurodegenerative diseases, all of which are characterized by unwanted cell loss with an inflammatory component. In addition, necroptosis is presumed to show protective effects against certain types of malignancies and improve disease outcomes [32,33,34]. 

Considering the potential role of necroptosis in a number of human pathological diseases, necroptosis has emerged as one of the most advanced intriguing mechanism-based therapeutics. In this review, we aim to highlight the molecular mechanism of necroptosis and discuss the potential intricacies of necroptosis machinery for possible clinical interventions. Here, we mainly emphasize the function, activation, and regulatory mechanisms of the necroptotic molecules (RIPK1, RIPK3, and MLKL). Additionally, we provide an overview of recent therapeutic strategies, drugs, and non-coding RNAs (ncRNAs), which provide a novel therapeutic perspective for the regulation of necroptosis. 

## 2. General Mechanism of Necroptosis 

The signaling of necroptosis is mainly stimulated through ligand binding to death receptors (DRs), and the action of intracellular RIPs family kinases that assemble into complexes are either associated with apoptosis or necroptosis. 

### 2.1. Necroptosis Signaling: Necrosome Formation 

Among the wide range of stimuli, TNF-α/TNFR1-stimulated necroptosis is the most common and well-understood signaling mechanism [23]. TNF-α is a key pro-inflammatory cytokine that is known to be associated with multiple inflammatory diseases, including cancer. Following the binding of TNF-α with TNFR1, subsequently, receptor trimerization leads to the requirement of multiple proteins, namely RIPK1, TNFR-associated death domain (TRADD), TNFR-associated factor 2 (TRAF2), TRAF5, and cellular inhibitor of apoptosis 1 (cIAP1), cIAP2, and linear ubiquitin chain assembly complex (LUBAC) toward the intracellular domains of TNFR1, which leads to rapid formation of membrane-associated complex I [35]. In this complex, TRADD functions as an adaptor molecule for the recruitment of RIPK1 to TNFR1. The linear and Lysine-63-linked ubiquitinylation of RIPK1 intermediate domains (ID) are mediated by LUBAC and cIAP1/2, respectively [36]. This poly-ubiquitinylation of RIPK1-ID further recruits and activates the transforming growth factor-activated kinase complex (TAK1 and TAK1-binding protein (TAB) 1/2) and the inhibitor of the NF-κB kinase (IKK) complex (consisting of NF-κB essential modulator (NEMO), IKKα and IKKβ) [37,38]. Afterward, both IKK and TAK complexes lead to activation of pro-survival NF-κB and MAPKinases signaling pathways [23], which further transactivate cytoprotective genes, such as cellular FLICE-like protein (cFLIP), and stimulate cell survival [36]. Hence, complex I is an essential checkpoint for cell survival and necroptosis. Furthermore, phosphorylation of RIPK1 at Ser25 by IKKα/β directly inhibits RIPK1 kinase activity and prevents the assembly of the death-inducing signaling complex (DISC), also known as complex-II [39,40].

However, complex-II is formed when there is absence or inhibition of cIAPs, TAK1, and NEMO activity (IAPs inhibitors treatment) due to translational inhibition (treatment of cycloheximide) or due to the activation of two deubiquitinases, cylindromatosis (CYLD) and A20, which hydrolyze Lys-63-linked ubiquitin chains and lead to deubiquitination of RIPK1 [41,42]. Two different types of complex-II (IIa and IIb) are formed that can be discriminated depending on their protein composition and activity. Within fractions after deubiquitinating RIPK1, the formation of complex-IIa has been seen after releasing TRADD and RIPK1 from the plasma membrane, which binds to Fas-associated protein with death domain (FADD), caspase-8 and cFLIP_L_ in the cytoplasm. This results in triggering caspase-8 activation and then induction of apoptosis through proteolytic cleavage of RIPK1 and RIPK3. In the case where cIAPs and TAK1 or IKKα/β complexes are inhibited/inactive, a complex-IIb, similar to complex-I, except for TRADD, is formed, wherein RIPK1 kinase activity is involved in inducing apoptosis via caspase-8 activation. Moquin et al. [43] reported that activated caspase-8 also promotes switching from apoptosis to cell survival by CYLD cleaving. Thus, complex-IIa-mediated death-induced signaling is independent of RIPK1-kinase activity, while complex-IIb-induced apoptosis occurs through RIPK1-kinase activity. 

**Figure 1:**
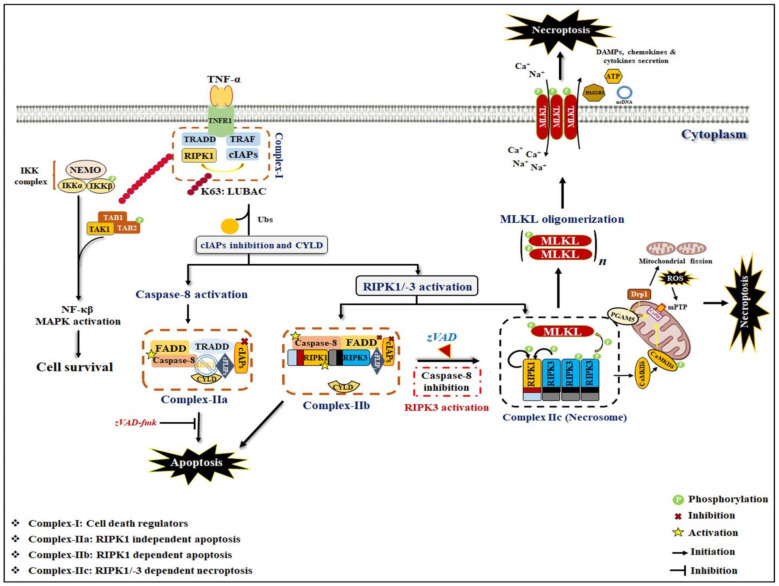
TNF-induced necroptosis signaling mechanism. Upon TNF binding, TNFR1 recruits numerous cell death signaling proteins such as TRADD, RIPK1, TRAF2, and cIAP1/2, assembling and formation of TNFR1 complex-I. The poly-ubiquitination of RIPK1 by cIAPs further activates the TAK and IKK complexes, which leads to the induction of NF-κβ and MAPKs pro-survival signaling pathways. Deubiquitination of RIPK1 by CYLD triggers the disassociation of TRADD and RIPK1 from TNFR1 and leads to the formation of either complex-IIa or -IIb. In complex-IIa, TRADD and RIPK1 are released from the plasma membrane and subsequently bind to FADD, caspase-8, and cFLIPL in the cytoplasm, resulting in caspase-8 activation and then induction of apoptosis through the proteolytic cleavage of RIPK1 and -3. In the inhibition/absence of cIAPs and TAK1 or IKKα/β complexes, a complex-IIb, similar to complex-IIa, except for TRADD, is formed, wherein RIPK1-kinase activity is involved in apoptosis induction through caspase-8 activation. When caspase-8 is inactivated/inhibited due to the pharmacological or genetic knockout, complex-IIc/necrosome is formed, and RIPK1/-3-dependent necroptosis is induced. In the necrosome complex, RIPK3 phosphorylates MLKL, and after phosphorylation, MLKL oligomerizes and translocates to the membrane. which leads to pore formation and release of DAMPs and pro-inflammatory cytokines. In addition, RIPK3 phosphorylates PGAM5 that in turn activates Drp1, which results in excessive mitochondrial fission that occurs under higher oxidative stress conditions, which further deteriorates the necroptosis process.

When caspase-8 is inactivated/inhibited due to pharmacological or genetic knockout, RIP kinases cleavage is stopped, and at the same time, expression of RIPK3 and MLKL is sufficiently high, resulting in transition of cell death pathways to necroptosis. During the necroptosis process, the first RIP1 kinase activates through the autophosphorylation of Ser161 residue at its N-terminal. After activation, RIPK1 recruits and binds with RIPK3 through their respective RIP homotypic interaction motif (RHIM) domains, which further lead to the development of a functional heterodimeric amyloid structure, also known as necrosome. Mitochondrial reactive oxygen species (ROS) was involved in activating RIPK1 through autophosphorylation, leading to the recruitment of RIPK3 and the formation of the necrosome complex [44]. Thus, ROS generation acts as a positive feedback circuit where necroptosis is maybe formed effectively. In addition, CYLD was also found to promote necrosome formation and activation by maintaining RIPK1 in a deubiquitylated state until the necrosome complex is not formed [43]. Upon activation, RIPK3 binds to its well-characterized substrate MLKL as a key mediator in necroptosis induction and promotes its autophosphorylation on Thr357/Ser358 residues in the activation loop. Usually, MLKL is present in cytosol in a monomer form, and after phosphorylation, it is converted into the oligomerized form, which subsequently translocates to the plasma membrane. However, MLKL oligomerization proceeds into necroptosis in two ways: (1) either it stabilizes the TRPM7 cation channel protein to stimulate more Na^+^ ion or Ca^++^ ion influx or (2) it increases the non-specific pore formations in the cellular membrane due to interacting with phosphatidylinositol phosphate and cardiolipin [24,28]. In addition, the RIPK1/RIPK3/MLKL necrosome complex shuttles to the mitochondrial outer membrane, and after phosphorylation, RIPK3 activates a mitochondrial phosphatase-phosphoglycerate mutase 5 (PGAM5), which is further involved in the induction of necroptosis by activation of dynamin-related protein 1 (Drp1), resulting in an accumulation of excessive dysfunctional mitochondria leading to excess ROS generation, which further accelerates the necroptosis process [25,26] (Figure 1).

### 2.2. Non-Canonical Necroptosis Signaling

**Figure 2:**
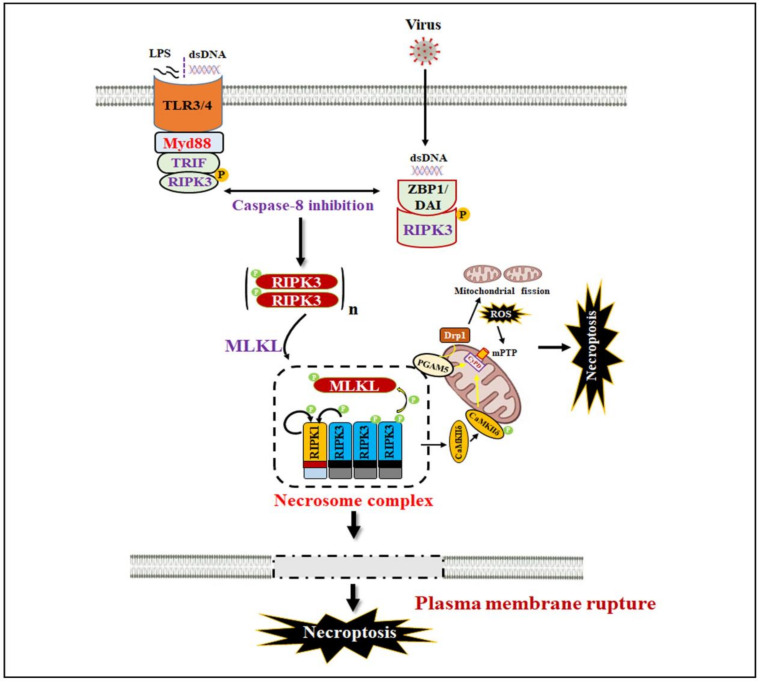
Necroptosis induction also occurs independently of death receptor pathways (referred to as non-canonical necroptosis). TLR3/-4 stimulation by LPS and dsRNA, respectively, leads to the recruitment of RHIM-mediated association of TLR adaptor molecules TRIF with RIPK3. The TRIF-RIPK3-MLKL necrosome functions independently of RIPK1. In addition, RIPK3-independent necroptosis is also stimulated by activation of cytosolic dsDNA sensor ZBP1/DAI. Upon binding of viral dsDNA, ZBP1/DAI undergoes a confirmation change that leads to its interaction with RIPK3. Further, RIPK3 after activation by TRIF and ZBP1/DAI phosphorylates itself and consequently MLKL to stimulate oligomerization and translocation to membranes, which leads to induced necroptosis features.

In addition to the TNF-α-mediated canonical necroptosis process, a non-canonical necroptosis pathway was also identified that is dependent on either the TLR3/4 receptor or the Z-DNA binding protein 1 (ZBP1)/DNA-dependent activator of IRFs (DAI) sensor. In non-canonical necroptosis, the necrosome complex is formed independently of RIPK1 kinase activity but is stimulated by RHIM-mediated interaction between TRIF and RIPK3 or ZBP1 and RIPK3 to promote assembly of the higher-order hybrid amyloid-like β-sheets structure. Numerous studies have described that the inhibition of RIPK1 in TLR3/4 receptor-mediated necroptosis does not impair this process [45,46]. The TRIF/RIPK3 and ZBP1/RIPK3 complex recruit and activate the downstream MLKL-induced signaling pathways, and as a result, higher ROS accumulates and induces necroptosis. Similar to canonical necroptosis, activated caspase-8 can also block TRIF-mediated necroptosis via cleavage of the TRIF-domain [47,48]. Unlike RIPK1, TRIF does not reside in protein kinase-like activity, but how TRIF activates the RIPK3 is poorly understood. It may be possible that RHIM-interacting domains can function as a scaffold protein, allowing for RIPK3 recruitment, autophosphorylation, and activation of MLKL, as was shown for ZBP1/DAI (Figure 2).

## 3. Role of a Necrosome Complex in Inflammation

Although apoptosis seems to be immunologically inactive, necroptosis is often more associated with robust inflammatory responses through the release of DAMPs either alone or in combination with pathogen-associated molecular patterns (PAMPs) into the extracellular space [49]. As a result, they facilitate inflammatory cell recruitment to the sites of infection, survey the extent of tissue injury, eradicate burst necroptotic bodies, and help in tissue remodeling and repair [50]. If necroptotic bodies cannot eradicate appropriately before cell lysis, the release of DAMPs would significantly enhance an inflammatory response wherein further releasing inflammatory factors can induce cell death, constituting a feed-forward loop. In particular, necroptosis stimulates the activation of myeloid lineage-specific (e.g., microglia and macrophage) cells, which enhances the levels of pro-inflammatory cytokines (e.g., IL-1 superfamily), thus triggering chronic and acute inflammatory diseases [51].

Furthermore, RIPK1 kinase activity and RIPK3 activation might also directly participate in the induction of inflammatory gene expression to induce inflammation independently of cell death [52]. Numerous studies have revealed that RIPK1 in L929 cells and mice models was associated with TNF-α production and secretion of JNK and NF-κβ-mediated pro-inflammatory cytokines during lipopolysaccharides (LPS)-induced inflammatory responses [52,53,54,55,56]. In other studies, it was reported that RIPK1 activation is essential in enhancing the level of IL1-β, IL6, IL12, and CXCL1 cytokines from macrophages via MAPKinase signaling (p38 and ERK1/2) activation following NF-κβ, SP1, and AP1 transcription factor activation, in the release of TNF from TNF-α treated cells through autocrine signaling, and is linked with neutrophilic dermatitis-mediated neuroinflammation in Ptpn6spin mice [54,57,58]. There are also mounting studies that RIPK1 activation can also mediate inflammatory responses independently from necroptosis in vivo. Kang et al. [59] reported that the activation of RIPK1 triggers a chronic inflammatory response in mice embryos when both apoptosis and necroptosis are absent. Lukens et al. [58] revealed that activation of RIPK1 spontaneously develops severe inflammatory diseases in SHIP-deficient mice through massive production of IL-1α cytokine. 

**Figure 3:**
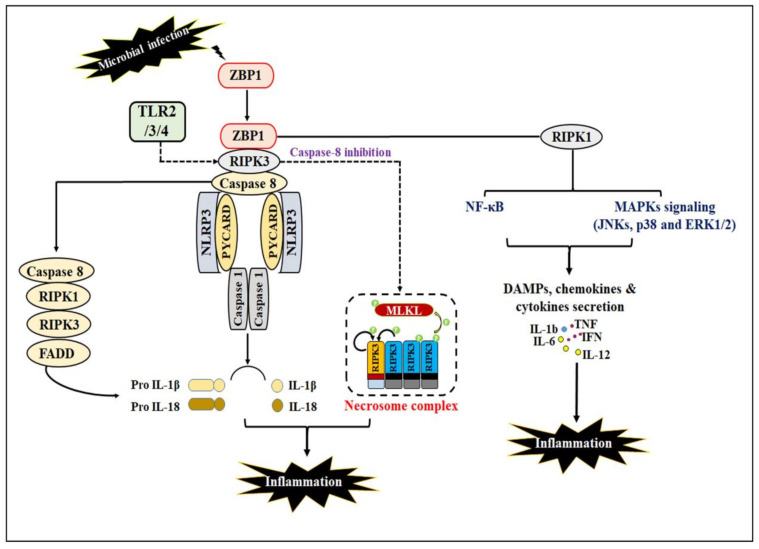
ZBP1 activation triggers the assembly of signaling complexes to be involved in the induction of necroptosis and inflammatory responses. Upon microbial infection or cellular stress response, activation of ZBP1 leads to the recruitment of receptor-interacting serine/threonine-protein kinase 3 (RIPK3) and caspase-8 to form cell death-signaling complexes. This ZBP1-RPK3-Caspase 8 scaffold further promotes the nucleotide-binding oligomerization domain-like receptor family pyrin domain-containing 3 (NLRP3). PYCARD and Caspase-1 contain the inflammasome complex, which leads to cleavage of pro-IL-1β and IL-18 into an active form, which results in inducing inflammatory responses. Activation of ZBP1 also stimulates RIPK1-driven inflammatory responses via NF-κB and MAPKs signaling (JNKs, p38 and ERK1/2) activation. Dotted lines also represent TLR2/3/4-induced inflammatory responses through RIPK3-MLKL, which induces necrosome complex formation under caspase-8 inhibition.

Despite RIPK1, RIPK3 also participates in immune regulation response by activation of NRLP3 inflammasome complex (NODD, LRR, and pyrin) formation. Upon microbial infection or cellular stress response, activated NLRP3 inflammasomes stimulate assembly of adaptor protein PYCARD (PYD And CARD Domain Containing) and subsequently activate caspase-1, leading to cleavage of pro-IL-1β and IL-18 into an active form. A growing number of studies revealed that activation of RIPK3 promotes cytokine production and inflammasome activation during TLR4-induced inflammatory responses, which occur under cell death deficiency or absence [60,61,62,63]. Numerous studies have shown that both the RIPK3 activity and MLKL are required for NLRP3-dependent inflammation [60,64,65], while some studies have reported that MLKL deficiency did not inhibit RIPK3-dependent cytokines production as well as maturation and inflammasome activation [60]. Few studies have reported that caspase-8 regulates the formation and activation of RIPK3-mediated NLRP3 inflammasomes in response to TLR2-TLR4 stimulation [62]. However, the precise mechanism of how caspase-8 functions in the regulation of RIPK3-dependent NLRP3 inflammasomes is still not known, but the above studies hypothesize that caspase-8 promotes the assembly and formation of a complex comprising RIPK1, RIPK3, FADD, and caspase-8 [21,60,62,66], and subsequently stimulates IL-1β maturation [66]. In contrast, few studies have shown that caspase-8 plays an inhibitory role via attenuating RIPK3-MLKL-mediated formation and activation of the NLRP3 inflammasome [67,68] (Figure 3).

## 4. Necroptosis: A Hallmark of Human Pathological Diseases

**Figure 4:**
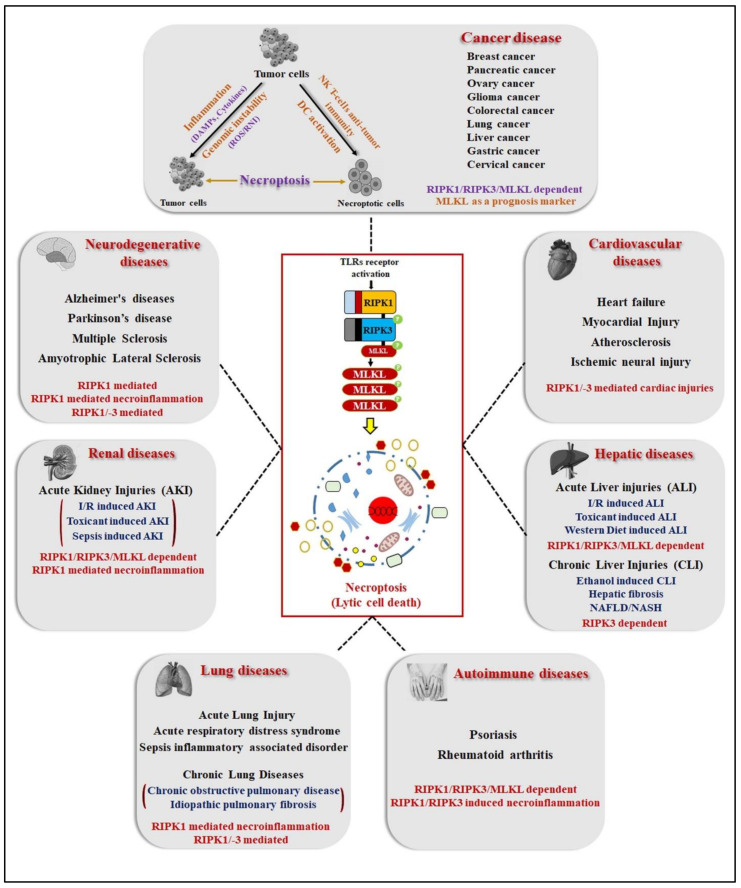
Potential role of necroptosis in human pathological diseases. The necroptosis pathway and its regulatory proteins (RIPK1/-3/MLKL) have been involved in numerous clinical diseases such as cancer, cardiovascular, lung, renal, hepatic, neurodegenerative, and inflammatory diseases. RIPK1, RIPK3, and MLKL-dependent necroptotic cell death have been observed in diseases such as acute kidney and liver injury, autoimmune disease, and cancer. In chronic kidney and lung injury and cardiovascular diseases, RIPK3/MLKL-dependent, but RIPK1-independent, phenotypes have been observed. Furthermore, RIPK1-mediated necroinflammation, independent of necroptosis, has been implicated in neurodegenerative, kidney, renal, sepsis, and autoimmune diseases.

The activation of RIPK1 and/or the RIPK3/MLKL complex as a mediator of cell death and inflammation has been involved in the pathogenesis of several human diseases. Numerous studies from the last two decades have been reported regarding both up- and downregulation of necroptotic proteins expression, and dysregulation of the transition of the apoptosis–necroptosis pathway have altered immunological consequences during cell death that significantly contribute to the development of neurodegenerative disorders, autoimmune disorders, infectious disease, and immune surveillance of cancers. Remarkably, the inhibition of regulated necrosis in various in vitro and in vivo models of human pathological diseases demonstrates the advantages of blocking necroptosis. This has developed more interest for researchers in this field, as there is thus far no development of effective therapeutic strategies for the cure of human diseases due to the lack of disease-specific target inhibitors. The following section will highlight the recently published research/clinical studies evident in the role of necroptosis in advanced and established human pathological diseases (Figure 4 and Table 2).

### 4.1. Necroptosis in Neurodegenerative Diseases (ND)

Neurodegeneration is a condition that includes the progressive loss of neurons’ structure or functions in specific areas of the brain. The exact causes for the vast majority of human ND are unclear, but neurons and synaptic loss are the last steps that could be amenable to clinical mediation. Accumulative evidence suggests that necroptosis and its associated necroinflammation have been involved by genetic, pharmacological, and histological approaches in ND, including multiple sclerosis (MS), amyotrophic lateral sclerosis (ALS), Alzheimer’s diseases (AD), Parkinson’s disease (PD) and Huntington disease (HD). Remarkably, the administration of Nec-1 provides neuroprotective effects against excitotoxicity as well as acute and chronic neurological injuries in numerous in vitro models and animal models, suggesting a mechanistic role of RIPK1 inhibitors in ND. However, RIPK1 inhibitors are also expected to be effective in curing other types of human ND diseases; the possibility of developing particular RIPK1 inhibitors that can cross the blood–brain barrier (BBB) provides a special opportunity for the treatment of ND. 

MS is an inflammatory disease of the central nervous system (CNS) that is characterized by the loss of oligodendrocyte structure or function and demyelination. In CNS lesions of both human patients and mouse models of MS, activated RIPK1 is re-localized to insoluble fractions, stimulating both inflammatory and cell death responses in macrophages and microglial cells [69]. Mechanistically, RIPK1 is highly expressed in microglia and macrophage cells in both human and mouse brain samples, and its inhibition may dampen inflammatory responses and recover the phagocytic capacity of these types of cells [146]. Additionally, other cells in the CNS exhibited higher expression of RIPK1 and its downstream signaling molecules (Caspase-8, RIPK3, and MLKL), which were also increased in the postmortem brain tissues of MS patients. Ofengeim et al. [69] identified that oral exposure of cuprizone to wild-type mice led to oligodendrocyte degeneration and demyelination in the corpus callosum, characterized by activation of RIPK1/RIPK3-dependent signaling without caspase-8 activation. Moreover, by performing immunostaining assays, the same groups could detect a higher level of RIPK1 and oligomeric amyloid-like structure induced by cuprizone. These findings suggest that formation of an oligomeric amyloid-like structure activates RIPK1/RIPK3-dependent signaling by stimulating their association. Exactly how RIPK1/RIPK3-kinase activity within these amyloid-like structures affects the surrounding extracellular environment (including aggregation and misfolding of other protein) still is unknown. Tissue samples from the CNS lesions of MS patients showed increased expression of FLIPL (a long-form of FLIP), an inhibitor of caspase-8 activation, which might stimulate the cleavage of caspase-8 to produce a p43 subunit but blocking full caspase-8 activation and by subsequently inducing the activation of necroptosis [69]. Under normal conditions, FLIPL is expressed in the many cell types of the CNS, including microglia and a precursor and mature oligodendrocytes, but to a lower extent in neurons [70]. Likewise, in EAE (experimental allergic encephalomyelitis) models of MS, ongoing inflammatory responses via NF-κβ pathway activation might promote FLIPL expression, which eventually facilitates necroptosis through defective caspase-8 activation [71,72]. Therefore, caspase-8 inactivation may provide an important mechanism for sensitizing cells to the necroptosis in progressive MS. Earlier studies had shown that TNF/TNFR1-mediated necroptotic neuronal and oligodendrocyte losses were associated with the increased expression of RIPK1 and phosphorylation levels of RIPK3/MLKL in in vitro and in vivo MS models, rather than apoptotic signaling [69,73]. Further, use of RIPK1-inhibitor Nec-1s or genetic deletion of RIPK3/MLKL could ameliorate TNF/TNFR1-induced neuronal and oligodendrocyte death in MS models, suggesting a functional link between necroptosis and neurodegeneration in MS-like pathology, and thus, necroptosis signaling holds great promise for therapeutic intervention. 

ALS is a progressive neurodegenerative disorder characterized by degeneration of motor neurons (MN) in the brain and spinal cord, leading to muscle atrophy and eventual paralysis. Re et al. [147], for the first time, reported that the mechanisms underlying loss of MN degeneration in ALS were dependent on RIPK1/RIPK3/MLKL-mediated necroptosis but not on caspase-dependent pathways. They found that targeting these necroptotic proteins through genetic and pharmacological interventions using RIPK1/RIPK3/MLKL shRNA, Nec-1, and NSA ameliorated MN degeneration and improved motor performance, supporting the role of necroptosis in ALS pathogenesis. Further, Ito et al. [74] defined the mechanisms of MN degeneration in ALS by mutations in optineurin (optn) and TBK1 genes, which are implicated in both familial and sporadic forms of ALS. Optn is a ubiquitin-binding protein that mediates K48-ubiquitinylation and the proteasomal degradation of RIPK1. Optineurin-deficient (optn^−/−^) mice developed neuropathology resembling ALS, due to a higher level of RIPK1, RIPK3, and pMLKL and necroptosis in the spinal cord of optn^−/−^ mice, which was rescued by inactivating RIPK1-kinase activity. Demyelination and axonal degeneration in the spinal cord of optn^−/−^ ALS mice were obstructed in both RIPK1- and RIPK3-deficient mice or were prevented by Nec-1s-mediated inhibition of RIPK1, thus showing improvements in motor performance, survival, and axonal pathology. It has also been shown that *optn* prevented MN demise from TNF-α-induced apoptosis via interactions with caspase-8 at its death effector domains to block the recruitment of FADD and downstream caspase activation. Moreover, RIPK1 deficiency/inhibition by nec-1s or RIPK3 genetic silencing protects against the demise of MN and astrocytes and delays the onset of motor dysfunction in the spinal cord of SOD1^G93A^-mutated mice models of ALS. Overall, these results suggest the roles of RIPK1 and necroptosis in the mechanisms of axon demise and neuroinflammation in ALS [74,75,76].

TBK1 mutations are major genetic factors in patients with ALS/frontotemporal dementia (FTD) disorder, and to a lesser cause, in ALS patients alone. TBK1 knockout mice (TBK1^−/−^) showed increased RIPK1 levels as a result of embryonic lethality, which are rescued by genetic/pharmacological inactivation of RIPK1 [77]. This observation is consistent with in vitro studies wherein TBK1^−/−^ cells were more sensitized to die by TNF-α-induced necroptosis, which was prevented by nec-1s [77,78]. Based on these observations, further groups also found that TBK1 directly regulates RIPK1 activity through phosphorylation at Thr189 to suppress RIPK1 kinase activity. Furthermore, the haploid loss of TAK1 expression, another suppressor of RIPK1, in a myeloid lineage of TBK1^+/−^ mice, leads to late-onset ALS/FTD-like pathology facilitated via diminished RIPK1 inhibition. 

AD is the most common progressive neurodegenerative disorder marked by a cognitive impairment with loss of memory and behavioral changes. It is characterized by the presence of amyloid-β (Aß) plaques and neurofibrillary tangles, which are composed of hyperphosphorylated tau proteins in extracellular and intracellular neural tissues, respectively. Loss of synaptic function and cholinergic neurons are prominent features of this disorder. Insoluble fractions of human postmortem brain samples from patients with late-stage AD showed a higher level of RIPK1, RIPK3, and MLKL as compared with standard age-matched brain samples and positively correlated with the reduction of brain weights and Braak stages [79,80]. Further, using the 5xFAD mice model, Caccamo et al. [79] detected an increased level of necrosome machinery markers in the brains of these mice models compared to non-transgenic mice. Treatment with RIPK1 inhibitors nec-1s decreased the pMLKL/MLKL ratio and prevented neuronal loss, supporting the role of necroptosis in the mechanism of neuronal death in this model. In a recently published study, the expression of RIPK1, RIPK3, and MLKL, at both mRNA and protein levels, was restricted to the granulovacuolar degenerative lesions in degenerating neurons of AD and in preclinical stages of AD pathology [81]. Earlier studies have reported that RIPK1 plays a key role in mediating neuroinflammation in AD [80,148]. The activation of RIPK1 in microglia was shown to drive the transcription of innate immune response-related genes. In particular, expression of *Cst7, Ch25h, Csf1*, and *Clec7a* is increased in microglia of various mice models of ALS and AD, for example, SOD1G93A and 5xFAD mice, and in aging microglia. RIPK1 activation has been shown to regulate the expression of the *Ch25h* (cholesterol 25-hydroxylase) gene, one of the lipid metabolism-related genes, in microglia of two animal models of AD, and it positively correlates with Braak staging of the disease. Further, Nec-1 administration suppresses *Ch25h* gene expression in microglia of AD mice models, leading to reduced neuroinflammation and cognitive impairments, suggesting that altered lipid metabolism might be associated with AD pathogenesis [149]. Additionally, for *Ch25h*, RIPK1 regulates the expression of *Cst7, Clec7a,* and *Csf1*, which have been recently shown as biomarkers for disease-associated microglia (DMA) located in spatial proximity to Aβ-plaques in both AD postmortem brain samples and mice models. Upregulated *Cst7*, which encodes a lysosomal/endosomal cathepsin inhibitor named cystatin-F, was found in microglia around deposits of Aβ-plaques in an APP/PS1 AD mice model, while dispersed microglia were negative for *Cst7* expression. Higher expression of *Cst7* may lead to lysosomal dysfunction in microglia and may promote diminished proteostasis in AD. Further, RIPK1 inhibition suppressed microglial *Cst7* expression in an APP/PS1 AD mice model, enhancing the uptake and degradation ability of Aβ-plaques by microglia in AD mice models [80,82]. These observations envisage that inhibition of RIPK1 activity in AD patients should lead to reduced inflammation in microglia, accumulation of Aβ-plaques, and restore the phagocytic activity of DAM. Asanomi et al. [150] identified a rare non-synonymous SHARPIN variant associated with an increased risk of late-onset AD (LOAD) in Japanese people. Burger et al. [83] reported that *cpdm* mice with SHARPIN deficiency induced TNF-dependent severe dermatitis and multi-organ inflammation that could be attenuated by RIPK1 inhibition. Thus, suppression of SHARPIN activity might contribute to the possibility of developing LOAD by attenuating M1 ubiquitination of RIPK1 to stimulate its activation. 

PD is the second most common neurodegenerative disorder marked by loss of dopaminergic neurons (DN) in the mid-region of the brain, especially substantia nigra. In postmortem substantia nigra samples of patients with PD, a profound activation of RIPK1, RIPK3, and MLKL in conjunction with defective mitochondria and lysosomes has been observed. Furthermore, using the systemic RNAi screening approach, Amin et al. (2018) identified a leucine-rich repeat kinase 2 (LRRK2) gene, which is often mutated in PD, and which shown to induce RIPK1-dependent apoptosis by activation of Complex I-associated RIPK1. In addition, Optic atrophy 1 (OPA1), a GTPase that regulates mitochondrial homeostasis (fusion and fission), is mutated in some PD patients. Dopaminergic neuronal cells-derived induce pluripotent stem cells from OPA1-mutant PD patients showed severe mitochondria deformity with cell death that was rescued by treatment with Nec-1s [84]. In addition, established in vitro and in vivo PD models also suggest that treatment of Nec-1s can rescue dopaminergic neuron loss in PC12 cells and mice exposed with 6-hydroxydopamine (6-OHDA) and MPTP, respectively, raising the possibility that activation of necroptotic machinery might promote neuronal demise [84,85]. In addition, Liu et al. [86] found that higher RIPK1 activation in MPTP-exposed SH-SY5Y cells and mice causes neurotoxic effects through activating the ASK1/JNK signaling pathway, while the pretreatment of Nec-1s showed opposite effects. Further, these studies also suggest that a higher level of reactive oxygen species (ROS) due to mitochondrial dysfunction might be directly responsible for RIPK1 activation in PD and other neurodegenerative disorders. 

### 4.2. In Renal Diseases

Several studies have observed the role of necroptosis (e.g., higher RIPK1, RIPK3, and MLKL expression and/or phosphorylation levels) in different forms of kidney disease [151,152,153]. RIPK1 kinase-deficient (KD) mice, RIPK3^−/−^ mice, and/or treatment with Nec-1 all showed nephroprotective effects in almost all forms of kidney injuries, which confirms that necroptosis plays a critical role in the development of renal diseases. 

Acute kidney injury (AKI) is a rapid reduction of renal functions related to an increase in serum creatinine level, declined excretory functions, and accumulation of waste toxic metabolites. In ischemia/reperfusion (I/R)-induced AKI models, increased levels of necroptotic markers (RIPK1, RIPK3, and MLKL) were observed in the renal–proximal tubular epithelial cells [154,155,156]. Corroborating to this observation, RIPK3^−/−^ or RIPK1 KD mice are less sensitive to I/R renal injury compared to wild-type mice, which relates to limited renal tubular injury, plasma creatinine level, and long-term inflammatory cell influx. MLKL^−/−^ mice shows a more protective efficiency against renal I/R injury than the effect of RIPK3^−/−^ mice [157], and thus, this finding may be proposed that RIPK3 can independently aggravate kidney injury compared to MLKL and other necroptotic molecules. Moreover, RIPK3^−/−^ and MLKL^−/−^ also show reduced NRLP3 inflammasome activation and IL-1β secretion during I/R response, which implies that a “necroinflammatory” cycle is involved in the reciprocal stimulation of both necroptosis and NLRP3 inflammasome activation and possibly underlies the transition from AKI to chronic kidney disease (CKD) [97]. In addition, blocking DAMPs (e.g., HMGB1) and cytokines secretion through neutralizing antibodies either before or after I/R injury reduced TLR-4-mediated necroptosis and improved renal functions [158]. Mitochondrial dysfunctions in I/R renal pathology suggest that the role of mitochondrial membrane permeability transition (mPT), and cyclophilin D (CypD) and peptidyl-prolyl cis-trans isomerase F (PPIF), a key mediator of mPT, are importantly involved in I/R-mediating renal injury [87,88]. However, genetic deficiency of these mPT regulatory proteins partially protects against I/R renal injury. CypD/RIPK3 double-knockout mice with the combination of Nec-1s and sangliferin (mPT pore inhibitor) markedly protect against renal I/R injury [152]. This observation suggests that RIPK3 and mPT-dependent necroptosis are both involved in I/R renal injury. This study showed that the nephroprotective effects of Nec-1 and RIPK3 ablation are independent of CypD in the I/R renal model. Along with similar findings, the RIPK3^−/−^ genotype also confers significant protection against mild I/R injury symptoms, and this protection can be extended to severe I/R by the concomitant deletion of PPIF. Further, similar results were obtained by using a combination of Nec-1, sangiferin, and ferrostatin-1 (Fer-1) (an inhibitor of ferroptosis), suggesting that other forms of regulated necrotic cell death are also involved in the pathophysiology of AKI [89,152]. 

In a folic acid-induced AKI model (FA-AKI), higher levels of RIPK3 and MLKL were found in renal tissues; however, RIPK3 or MLKL deficiency did not preserve renal function early points (48 h). Administration of Fer-1, but not a Nec-1, reduced renal injury, oxidative stress, and tubular cell death in this model. During the late stages of FA-AKI, it was observed that persistent renal dysfunction reliant on intact TWEAK-Fn14 axis mediated necroptosis. Further, this was suppressed by Nec-1 or a genetic knockout of RIPK3 or MLKL, which suggests that inflammation-dependent necroptosis can aggravate AKI in the later stages [90,91]. Moreover, RIPK3 or MLKL genetic ablation deteriorates cisplatin- and oxalate crystals-driven nephrotoxicity [92,93]; however, this genetic deletion could only partially mitigate renal toxicity [92] and nephrotoxic nephritis [29] and did not sufficiently reduce cell death, which was observed by TUNEL staining, indicating that additional pathways may be involved in such types of nephrotoxicity. In this line, CypD/RIPK3 double-knockout mice displayed improved renal functions and prolonged survival in comparison with the RIPK3^−/−^ mice, indicating that mPT-mediated necroptosis may play an indispensable role in cisplatin-induced renal toxicity [94]. Further, Ning et al. [159] found that Nec-1 administration significantly alleviates cisplatin-mediated nephrotoxicity in C57BL/6 mice through exerting anti-necroptotic, anti-apoptotic, anti-oxidative stress and anti-inflammatory effects, and up-regulates autophagy-associated protein levels. Some studies demonstrate that Z-VAD-fmk can increase nephroprotective efficacy of Nec-1 in cisplatin-induced AKI models [160,161]; in contrast, others reported that Z-VAD-fmk shows no more protective effects in mice experience to I/R-driven or iodinated radiocontrast-induced AKI [151,152]. This discrepancy suggests that apoptosis also contributes to renal toxicity by cisplatin and necroptosis in others. 

In a sepsis-induced AKI model, mice subjected to CLP surgery showed elevated levels of pRIPK3, in addition to this RIPK3, which were observed in renal tubular epithelial cells [96]. RIPK3^−/−^, but not MLKL^−/−^, mice show more resistance to renal injury than RIPK3^+/+^ mice after CLP [96]. In this line, RIPK3 deficient-mediated nephroprotective effects were associated with decreased oxidative stress and mitochondrial dysfunction via inhibiting the translocation of NOX4 in mitochondria and upregulation of mitochondrial Complex I and III expressions. Consistently, NOX4 deletion mice exhibited markedly reduced renal injury in a sepsis-induced AKI model [96]. This finding indicates that RIPK3 may aggravate renal injury in sepsis-induced AKI via disrupting mitochondrial functions and may represent a promising therapeutic marker of human sepsis-induced AKI. Liu et al. [162] demonstrated that Rgmb-KO mice are more prone to I/R injury and oxalate renal toxicity than wild-type mice, and when treated with Nec-1 or GSK’963, a necroptosis inhibitor, markedly reduced cell death in renal proximal tubular cells and preserved kidney functions in this mice model. Rgmb inhibited tubular cell necroptosis specifically through reduced MLKL membrane association during AKI.

In addition to AKI, necroptosis has also been implicated in the pathophysiology of renal fibrosis, as a common pathogenic response to injury in chronic kidney disease (CKD) [95]. In unilateral ureteral obstruction (UUO) and an adenine diet kidney fibrosis model (ADKFM), RIPK3 was upregulated in kidney tubular epithelial cells. Moreover, in UUO and AD fibrosis models, renal fibrosis was reduced, while renal function was improved in RIPK3^−/−^, but not in MLKL^−/−^ mice versus WT mice. RIPK3 exacerbates renal fibrosis specifically via the PI3K/AKT-dependent activation of metabolic enzyme ATP citrate lyase (ACL). Further, pharmacological inhibition of ACL activity suppressed RIPK3-mediated kidney fibrosis in the UUO model, which suggests that RIPK3 is involved in metabolic regulation and may present as a promising therapeutic target in human CKD [95]. Additionally, Chen et al. [97] demonstrated that in the RIPK3^−/−^ and MLKL^−/−^ system, necroptosis not only reduces in tubulointerstitial cells, but it also blocks infiltration of the inflammatory cells and renal fibrotic remodeling. This author also reports that the recruitment of inflammatory cells (T-lymphocytes cells and macrophages) triggers more necroinflammation and IL-1β secretion, and lastly, promotes interstitial fibrogenesis for the long term after I/R injury. Therefore, these studies indicate that necroinflammation is stimulated by RIPK3/MLKL-dependent necroptosis and plays a crucial role in the progression of I/R injury to CKD. 

### 4.3. In Hepatic Diseases

Necroptosis has been implicated in acute or chronic liver failure and acts as a modulator of major liver diseases, such as non-alcoholic fatty liver disease (NAFLD), alcoholic liver disease (ALD), non-alcoholic steatohepatitis (NASH), and toxic hepatitis model [98,163,164]. However, how necroptosis promotes the pathophysiology of liver injury is unclear. 

Numerous animal models close to acute human liver disease have been developed. In mouse models of acetaminophen hepatotoxicity [163,165] and ethanol and dietary intoxication [99,165], concanavalin-A induced fulminant hepatitis [163], and NASH [101,102], an RIPK3^−/−^ genotype, or treated with dabrafenib (an inhibitor of the active kinase BRAF oncogene, which also suppresses RIPK3 kinase activity) [166], provides considerable hepatoprotective effects against liver injury in virtually of all these mouse models. Following this, mice with pre-treatment of Nec-1 exhibit more resistance against acetaminophen-induced hepatotoxicity than wild-type control mice. However, another study on acetaminophen toxicity in mice revealed no hepatoprotective effects of the RIPK3^−/−^ and MLKL^−/−^ genotype [167]. These contradicting findings argue that involvement of necroptosis is as a whole in the etiology of hepatic acetaminophen intoxication. Rather, it seems that RIPK1 supports acetaminophen hepatotoxicity independent of necroptosis. Saeed et al. [168] found that RIPK1, RIPK3, MLKL, and PGAM5 expressions were not changed in the hepatic I/R injury murine models. Furthermore, mice with RIPK3^−/−^ and pre-treatments of Nec-1s in WT mice did not show hepatoprotective effects in the liver I/R model. In contrast, Ni et al. [103] found that in the diet-induced hepatic steatosis model, RIPK3-KDKI or RIPK3-KO mice show protective effects against liver I/R injury in the late phase but not in the early phase, whereas MLKL^−/−^ mice were protected from hepatic I/R injury in both early and late phases. Furthermore, mice with specific RIPK1 deletion in liver parenchymal cells were found to be more sensitive to fulminant viral hepatitis [169]. Likewise, in another study, RIPK1 deficiency aggravated inflammation-mediated hepatic injury in mice, and treatment with Nec-1 even exacerbated liver injury, suggesting that RIPK3-dependent necroptosis plays a complex role in liver toxicity.

Necroptosis has also been intimately involved in the etiology of chronic liver injury. One of the most common reasons for chronic liver injury worldwide is excessive alcohol consumption. Roychowdhury et al. [98] found that higher RIPK3 expression was present within the liver of both ALD patients and the murine models of ALD. Subsequently, chronic ethanol- and Gao-binge (mimics chronic alcoholic injury model) ethanol-mediated liver injuries were attenuated in RIPK3-deficient mice as compared to WT control, but pre-treatment with Nec-1 had no effects, suggesting the involvement of RIPK3 in ethanol-induced liver injury and progression to ALD [99,100]. The author of this study also revealed that the expression of proteins related to the proteasome system (α-2 subunit and ATPase 1) were decreased in mice with Gao-binge treatment. When genetic or pharmacological inhibition of these proteins led to an accumulation of RIPK3 in mouse livers, the impaired proteasome function in the liver by chronic alcohol exposure can suggest a prominent role of RIPK3. However, no data are currently available on the possible role of MLKL in alcohol-induced liver injury. Moreover, in high fat diet (HFD) or methionine-choline deficient (MCD) diet-fed mice that serve as a model of NAFLD and NASH, expression of TNF-α, RIPK3, and MLKL were increased in the liver, which contributes to liver injury, fibrosis, and inflammation [101,102]. Wu et al. [105] found that in HFFC (high in fat, fructose, and cholesterol) diet-fed mice models, a higher pMLKL level, but independently of RIPK3, promotes liver injury, fibrosis, steatosis, and inflammatory responses through blocking autophagosome to lysosome fusion, and as a result, the autophagy flux was blocked. Thus, these studies suggests the profound involvement of RIPK3 and MLKL in hepatic steatosis liver injury.

### 4.4. In Pulmonary Diseases

Several experimental pieces of evidence have observed that the necroptosis pathway and its regulatory proteins contribute to the etiology of various pulmonary diseases and related disorders. An emerging paradigm is that RIPK3 is a central mediator of lung tissue injury in murine models of acute lung injury (ALI), sepsis/systemic inflammatory associated organ injury, and chronic lung diseases [170,171,172]. 

In a model of ventilator-induced acute lung injury (VILI), mice subjected to mechanical ventilation at high tidal volume (25 mL/kg), RIPK3 deficiency but not MLKL deficiency conferred resistance from lung inflammation and injury than their WT mice, suggesting that RIPK3-induced necroptosis is a crucial mechanism to augment inflammation and lung tissue injury [106]. The authors also reported that fatty acid β-oxidation (FAO) pathway inhibition-associated tissue injuries were significantly ameliorated in RIPK3 deletion mice, recommending a possible connection between RIPK3 signaling, mitochondria-mediated FAO, and tissue injury progression [106]. Corroborating this, RIPK3 deficiency or treatment with GSK’872, a specific inhibitor of RIPK3, markedly attenuates inflammatory cell influx along with IL-1α/β, IL-6, IL-18 secretion, and HMGB1 release, and tissue injury in an LPS-induced ALI model [97]. Moreover, a novel RIPK3/NRLP3 complex, which is generally involved in caspase-1 activation, IL-1α/β and IL-18 secretion, was observed in the THP-1 macrophages of an ATP- and LPS-induced ALI mice model, suggesting that RIPK3-mediated inflammasome activation and necroptosis processes are mutually co-regulated in the pathophysiology of lung injury [97]. In addition, pretreatment of Nec-1s exerted protective effects on an LPS-induced ALI mice model through remarkable suppression of necroptosis, inflammation, and ROS overgeneration, with increased cellular antioxidant capacity [108], thus suggesting that necroptosis inhibition may serve as an effective anti-inflammatory therapy to treat ALI. 

Acute respiratory distress syndrome (ARDS) is a type of respiratory failure defined by a large systemic inflammatory response leading to increased lung capillary permeability with subsequent fluid accumulation and fibrosis. Pan and colleagues [107] were the first to establish that treatment with Nec-1 or RIPK3 deficiency attenuated the hypothermia symptom, a systemic inflammatory response by decreasing neutrophil infiltration, and increased the survival rate of oleic acid-fed rat, which acts as a model of ARDS. Similarly, Wang et al. [109] found that in the LPS-induced ARDS mice model, RIPK3-deficient mice exhibited a reduction in hypothermia, improved survival, and reductions in systemic inflammation via blocking neutrophil infiltration and cytokines secretion in lung parenchyma tissues. 

A specific link between necroptosis and inflammation was observed in a TNF-α induced SIRS (sepsis/systemic inflammatory associated disorder) mouse model. Genetic ablation of RIPK1 and RIPK3 or treatment with Nec-1 provided significant protection against TNF-induced lethal SIRS and diminished circulating DAMPs level, suggesting the importance of both RIPK1 and RIPK3 in mediating cellular damage and mortality by necroptosis during TNF-induced SIRS [46]. In a similar model, RIPK1^D138N/D138N^chimeric mice reconstituted with WT mice-derived hematopoietic stem cells displayed considerable protection against TNF-induced SIRS, whereas its reconstitution with RIPK1-inactive hematopoietic cells was not protected against TNF-induced SIRS. These studies indicate necroptosis abrogation in non-hematopoietic cell lineages mediated for protection against TNF-induced SIRS in RIPK1-inactive mice [173]. RIPK3 deletion in mice also provides protection from CLP-induced polymicrobial sepsis, as shown by increased survival and declined circulating DAMPs level, proinflammatory cytokines secretion, and the reduction of neutrophil infiltration [46]. In a model of cecal slurry-induced neonatal sepsis, mice administrated with Nec-1 or RIPK3 deficiency exhibited resistance against systemic and pulmonary inflammation-mediated lung injury and improved survival. These findings suggest that both RIPK1 and RIPK3 participate in a systemic inflammatory response in cecal slurry-induced neonatal sepsis [110,111]. 

Chronic obstructive pulmonary disease (COPD) is a chronic respiratory disease in which loss of alveolar surface area (emphysema) and airway inflammation (bronchitis) results as a consequence of chronic cigarette smoke (CS) exposure. Mizumura et al. [112] first demonstrated that necroptosis through RIPK3 activation promotes the pathogenesis of COPD in CS-exposed pulmonary epithelial cultured cells and that cell death response reduces in the presence of necroptosis inhibitor, Nec-1. The investigators also found that CS-induced cell death responses are associated with autophagy-mediated mitochondrial elimination in pulmonary epithelial cells. In CS-induced cellular injury, DRP1 mediated excessive mitochondrial fission and the activation of PINK1 mediated mitophagy, a selective autophagy for clearance of dysfunctional mitochondria, which indicates that mitochondrial dysfunctions might be associated with CS-induced cellular injury [112]. Further, genetic studies have shown that RIPK3 activation during CS exposure is integrated with PINK1-dependent mitophagy, in which genetic deficiencies of core autophagy (LC3B) and mitophagy proteins (PINK1) conferred protection to pulmonary epithelial cells against apoptosis and necroptotic cell death and against emphysema and bronchitis pathophysiology in vivo [113,114]. Thus, these findings suggest that CS induced both mitophagy and autophagy, exerting adverse pro-pathogenic effects in CS-exposed mice. A recent investigation in murine models has found that lung injury and inflammation are the primary contributors that intensify CS-induced COPD progression [115]. Lu et al. [115] have shown a higher expression of RIPK3 and MLKL in CS-exposed mice/COPD patients’ lungs that promotes inflammation, bronchitis, and emphysema in COPD. Concurrently, Chen et al. [116] observed that CSE-induced necroptosis (higher p-MLKL level) secretes HMGB1 along with an increase in TNF-α, IL-6, and IL-1β, resulting in inflammation and COPD progression in CS-exposed lung epithelial cells/organoids and mouse tissues. Another finding also observed that mitoROS-activated necroptosis signaling is involved in CS-induced inflammatory responses in mouse alveolar macrophages and BMDMs via the modulation of the NF-κβ signaling pathway [174]. Thus, all these findings suggest that targeting necroptosis (especially RIPK3 and its downstream effectors) and its induced inflammation may be effective therapeutic strategies for COPD patients.

Idiopathic pulmonary fibrosis (IPF) is a rare chronic respiratory disease characterized by the progressive thickening and stiffening of lung tissues associated with scarring of lungs [175]. As of yet, limited studies have observed the role of necroptosis in the etiology of IPF. In a bleomycin (BLM)-induced IPF model, increased RIPK3 expression was observed in alveolar epithelial cells (AEC). Further, the presence of Nec-1 and RIPK3 deficiency significantly attenuates DAMPs release (i.e., HMGB1 and IL-1β) and lung inflammation via inhibiting neutrophil infiltration in response to BLM. Thus, necroptosis activation and DAMPs release may promote IPF pathogenesis [117].

### 4.5. In Cardiovascular Diseases

The necroptotic pathway and its key regulators have been reported in different cardiovascular pathologies, such as chronic heart failure, aortic aneurysm, myocardial injury, ischemic neural injury, and stroke [176,177,178]. RIPK3^−/−^ mice compared with WT mice were protected from heart failure caused by I/R or doxorubicin (a chemotherapeutic cardiotoxic agent), and are associated with decreased necroptotic myocardium response [118] [119]. Experiments with cardiomyocyte culture cells report that cardiac injury effects on RIPK3 activation appear to be triggered via a CaMKII-mediated signaling pathway, resulting in mPT and in necroptosis, instead of MLKL activation. Consistently, both CaMKII- and PPIF-deficient mice were also protected from I/R and/or cardiotoxicant-induced cardiac injury [119,120,121]. These findings were further contradicted by Oerlemans et al. [122], which reported that the cardioprotective effects with the use of Nec-1 in mouse and pig models of I/R cardiac injury are mediated by a reduction of pMLKL level. However, it remains unclear whether such types of cardioprotective effects truly come from the inhibition of RIPK1/RIPK3 downstream mediators, such as MLKL-dependent necroptosis. Furthermore, combined treatment with Nec-1 and zVAD-FMK remarkably increased cardioprotection against I/R cardiac injury, also indicating the importance of apoptosis in cardiac I/R injury [179]. In these lines, RIPK3 activation and subsequent necroptosis in atheroma macrophages lead to local and systemic inflammatory responses associated with atherosclerosis in low-density lipoprotein (LDL) receptor- and apolipoprotein E (APOE)-deficient mice [123,124]. Thus, both RIPK3^−/−^-LDR^−/−^ and RIPK3^−/−^-APOE^−/−^ mice exhibit diminished arteriosclerotic lesions, inflammation (macrophages and neutrophils infiltration), and delayed mortality in the late stages of disease progression versus LDR^−/−^ or APOE^−/−^ mice, respectively. Finally, in the elastase-induced abdominal aortic aneurysm model, mice with RIPK3^−/−^ or RIPK3^+/−^ exhibited more resistance to aneurysm growth and TNF-mediated inflammatory responses in cardiac smooth muscle cells [125]. Transplantation of RIPK3^+/−^ into a similar model of WT mice involves less lymphocyte filtration and DAMPs release in circulation and in more prolonged graft survival [126]. 

There is also evidence of necroptotic pathway activation and increased RIPK1 and RIPK3 expression in pathologies of myocardial infarction progression in response to myocardial ischemia (MI) and hypoxia, due to which impaired cardiac functions and the increased incidences of cardiovascular mortality result. Under conditions of MI injury, activation of necroptosis in myocardial infardium tissues and other heart tissues was found to trigger inflammatory cells infiltration such as neutrophils, macrophages, and T-cells, and aggravated the production of large numbers of TNF-α. Earlier studies have reported that TNF-α induced cardiomyocyte necroptosis, which is followed via RIPK1, RIPK3, and MLKL-dependent necroptotic signaling activation [118,180]. In conditions of OGD injury (an in vitro MI mimicking condition), necroptotic cardiomyocytes and cardiac dysfunction occurred due to defective autophagy degradation and the persistence of higher necroptotic molecule expression, as further evidenced by an increase in propidium-iodide (PI)-positive cardiomyocytes numbers in a time-dependent manner [127]. In a rat model of permanent left anterior descending coronary artery (LAD) ligation (in vivo chronic MI condition), constitutive necroptosis activation occurred after a week of MI progression compared with Sham WT rats by upregulating RIPK1 and RIPK3 expression [127]. In addition, another molecular element also found to be involved in necroptosis regulation in cardiomyocytes is *Traf2*, which plays a key role in cardiomyocyte homeostasis and survival by attenuating programmed cell death. In *Traf2^−/−^* mice, cardiomyocyte death, cardiac dysfunction, and cardiac remodeling were observed via activation of necroptosis signaling concomitant with suppression of NF-κβ-mediated cell survival signaling [180].

Overall, these observations indicate that necroptosis appears to exert a crucial role in the pathophysiology of cardiovascular disease. Thus, the use of necroptosis inhibitors has potential as a cardioprotective agent in the experimental models of several cardiovascular diseases; however, further studies are warranted to better investigate molecular mechanisms and possible off-targets of these inhibitors. 

### 4.6. Autoimmune Diseases

Autoimmune disease is a condition caused by abnormal functions of the immune system in the body’s tissues. Earlier studies reported that necroptosis and its key regulators are associated with some common forms of autoimmune diseases by sterile inflammation: psoriasis, rheumatoid arthritis, inflammatory bowel disease, and systemic lupus erythematosus.

Psoriasis is a chronic inflammatory autoimmune skin disorder characterized by rounded erythematous, dry, red, or white plaques in skin. Inflammation-related cell demise in keratinocyte skin cells plays a critical role in developing regulated inflammatory cascades in psoriasis. Honda et al. [128] found that enhanced RIPK3 expression facilitates psoriatic inflammation by stimulating the secretion of neutrophil-recruited cytokines/chemokines in keratinocytes cells in vitro and Aldara-induced psoriasis mice models in a necroptosis-independent manner. Further, Saito et al. [129] observed the skin samples of patients with psoriatic lesions and found that psoriatic inflammation and enhanced cytokines/chemokines secretions in keratinocytes skin cells are associated with reduced RIPK1 expression. Moreover, they have shown that the downregulation of RIPK1 increased its sensitivity to TNF-related apoptotic cell death ligands, which is critical in the pathology of psoriasis. Recently, Duan et al. [130] reported that RIPK1, RIPK3, and MLKL expression and their localization were significantly increased in all layers of epidermal skin cells in vitro and in the IMQ-induced psoriasis mice model. Further, the pharmacological inhibition of RIPK1 and RIPK3 through Nec-1s and NSA, respectively, confirmed the role of necroptosis in inflammatory cascades in both in vitro and in vivo psoriasis models. Thus, these studies suggest that necroptotic players might represent a novel promising therapeutic target for necroptosis-activated psoriasis pathogenesis.

Rheumatoid arthritis (RA) is a devastating systemic autoimmune disorder characterized by synovial membrane inflammation. Earlier studies have found a significant increase in necroptotic molecule (RIPK1/RIPK3/MLKL) expression in the synovium of a collagen-induced arthritis mice model. Furthermore, researchers also observed in arthritis mice models that Nec-1 administration significantly suppresses the inflammatory response and the secretion of TNFα, IL-17, IL-1β, and IL-6 cytokines, thus providing a protective effect in autoimmune arthritis [62,131]. Alternatively, Lee et al. [132] observed that IFN-γ reduced necroptosis via downregulated MLKL expression; in contrast, the reverse condition was found in IFN-γ^−/−^ mice. Therefore, these studies suggest necroptosis inhibition to be a promising approach for treating RA.

### 4.7. In Cancer Diseases

Cancer is a disease that is closely related to apoptosis, and resistance to cell demise is a key hallmark of cancer. In cancer, necroptosis has been recognized as both friend and foe; its dual roles in initiating and suppressing tumor growth have been reported in several forms of cancer. As a fail-safe form of cell death arising in cells wherein apoptosis fails to be induced, necroptosis can inhibit tumor growth. Nevertheless, similar to a necrotic cell death modality, necroptosis can activate inflammatory responses and can reportedly help in cancer growth, metastasis, and immunosuppression. 

#### 4.7.1. Necroptosis Defense against Cancer 

Numerous studies have suggested that necroptosis plays a significant role in maintaining the homeostasis and inhibition of tumor initiation, growth, and metastasis. Furthermore, necroptosis is a mechanism by which chemotherapeutic agents can eradicate cancer cells.

Dysregulation of key molecules in necroptosis signaling promotes tumor initiation and growth, suggesting that tumor cells can evade necroptosis to survive. Several studies have shown that RIPK3 protein expression is absent or reduced in two-thirds of 60+ cancer cell lines tested [34,181]. In addition, reduced RIPK3 expression was also found in clinical samples from human cancer patients, such as colorectal, breast, acute myeloid leukemia (AML), and melanoma. For example, Nugues et al. [133] found that RIPK3 expression is significantly decreased in AML cells, which resulted in the modulation of caspase-mediated apoptosis/necroptosis via p65/RelA cleavage and shifts toward NF-κβ to mediate cell survival. Similarly, another study also shows that knockout of RIPK3 in mice has markedly enhanced leukemogenesis by promoting the accumulation of leukemia-initiating cells and further connection between RIPK3 suppression and cell death blockage, which is also validated in primary AML patient cohorts [33]. Furthermore, downregulated RIPK1 and RIPK3 expression has been documented in colorectal cancer tissues versus normal tissues, and thus, their reduced expressions impair cancer cells’ responses to necroptosis stimulation [21]. Similarly, the low levels of RIPK3 and CYLD expression developed chronic lymphocytic leukemia (CLL) cells to resistance from TNF-α/zVAD-induced necroptosis [134]. In melanoma, the inhibition of CYLD expression through Snail, a transcription factor, promotes cell proliferation and invasiveness in vitro and cancer progression and metastasis ability in vivo [182]. In addition, low RIPK3 expression is associated with a worse prognosis in patients with breast cancer due to genomic methylation [34]. These findings implicate the anti-tumor and anti-inflammatory role of RIPK3 in cancer. Likewise, some reports point out that genomic methylation and/or hypoxia can play an oncogenic role in colorectal cancer cell lines via silencing of RIPK3 expression [183]. Further, McCormick et al. [135] found that lower expression of RIPK1 in head and neck squamous cell carcinoma is associated with disease progression. Similarly, Park et al. [136] reported that the expression of RIPK1, RIPK3, and MLKL is significantly downregulated in non-small cell lung cancer tissue (NSCLC) samples of a total of 253 NSCLC human patients (96 squamous cell carcinoma (SCC) cases and 157 adenocarcinomas (AC) cases); this downregulation was also positively correlated with worse prognosis. In addition, MLKL expression is significantly decreased in pancreatic adenocarcinomas and ovarian cancer tissues compared to the adjacent normal tissues, and this reduction was proven to be associated with disease progression [137,184]. 

Contrastingly, Wang et al. [174] showed that upregulation of RIPK3 expression reduces prostate cancer cells proliferation and tumorigenicity both in vitro and in vivo via phosphorylating MLKL, which was reversed by treatment of the MLKL inhibitor, suggesting that necroptosis induces cell death in prostate cancer cells. Collectively, these studies also suggest that downregulation of necroptotic molecule expression due to epigenetic/genetic changes during cancer progression allows cancer cells to evade anoikis, which may further stimulate tumorigenesis by increasing the metastatic capability of tumor cells. Further, some studies reported that higher RIPK1/3 expression may promote anti-metastatic effects in cancer cells via regulating redox-metabolism to kill the metastatic cancer cells [185,186]. Thus, enhanced RIPK1/3 levels generate excessive ROS production, and this higher ROS level might inhibit the metastatic ability of cancer cells. Furthermore, some reports are indicated that necroptosis also triggered immune activation against different tumors to constrain cancer progression. For example, Poly I:C (polyionisic:polycytidylic acid), a synthetic analog of viral dsRNA, induces TLR3/RIP3-dependent necroptosis in colorectal cancer and inhibits tumor growth by the activation of dendritic cells (DC)-mediated anti-tumor activity in vivo [138]. Similarly, Schmidt et al. [139] described that RIPK3 upregulation released interleukin-1α (IL1-α) from Poly I:C-induced necroptotic cervical cancer cells and activated DCs; these DCs then showed their anti-tumor effects by producing cytotoxic cytokine IL-12 or by activating CD8^+^ T-cells [187]. Werthmöller et al. [140] reported that treatment in combination of with the zVAD-fmk, a pan-caspase inhibitor that induces necroptosis, and other therapeutics, such as chemotherapy, radiotherapy, and hyperthermia, to B16 melanoma cells markedly inhibits tumor growth by decreasing infiltrated regulatory T-cells while increasing cytotoxic DCs as well as CD8^+^ T-cells infiltration around the tumor microenvironment. In addition, RIPK3 expression also induced natural killer (NK) T-cells-mediated immune response via the activation of mitochondrial PGAMF-NFAT/Drp1 signaling, independent of the necroptosis pathway, and that anti-tumor response of NK T-cells to metastatic cancer cells is diminished in RIPK3^−/−^ mice [141].

#### 4.7.2. Necroptosis in Cancer Progression

From the above section, it is easy to understand and accept the viewpoint that necroptosis plays an anti-tumor role in cancer. However, emerging research suggests that necrotic-programmed cell death promotes tumor progression, and numerous current studies showed that necroptosis is not involved in the progression of all cancer types.

It has been found that the expression of necroptotic molecules is commonly upregulated in some types of cancer, and it is highly correlated with malignancy and poor survival. For example, in glioblastoma cancer, RIPK1 expression is remarkably upregulated, and this upregulation is positively correlated with worse overall survival [143]. Likewise, RIPK1 expression is markedly upregulated in both clinical samples of lung cancer and cigarette smoke-exposed mice lung cancer models, and thus, RIPK1 has been suggested as a tumorigenic factor [144]. Furthermore, Liu et al. [142] reported that lack of RIPK1, RIPK3 and MLKL expression in breast cancer cells significantly lessens their proliferation and migration capacity in vitro and attenuates their ability to produce tumors in vivo. Moreover, in a xenograft model, the treatments of necroptotic inhibitor Nec-1s significantly delayed tumor growth. A similar study also found that a higher level of phosphorylated MLKL was linked with a worse prognosis and shorter survival in human patients with esophageal and colorectal cancer, suggesting that necroptotic genes play a key role in cancer progression. Further, Seifert et al. [145] found that RIPK1, FADD, RIPK3, and MLKL expressions were significantly increased in pancreatic ductal adenocarcinomas (PDA), which is accompanied by accelerated tumorigenesis. In addition, higher expression of RIPK1 was found to be linked with the higher metastasis rates in melanoma and confers a worse prognosis in melanoma potentially due to NF-κß mediated stimulation of tumor cells proliferation [188]. Using tissue microarray data of stage-I NSCLC samples from 394 patients, Lim et al. [189] found that the lower expression of RIPK3 and PELI1 with a combination of higher p53 expression, a key DNA damage response factor, is linked with poor survival of stage-I NSCLC patients. 

Instead of the role of necroptosis in the development and amplification of anti-tumor immunity, multiple studies have demonstrated that programmed necrosis/necroptosis may also involve recruiting immune inflammatory cells around the tumor microenvironment to promote tumor progression by fostering tumor cell proliferation, angiogenesis, and metastasis. Seifert and colleagues [145] have observed that necroptosis stimulates pancreatic oncogenesis by inducing CXCL1 (chemokine (C-X-C motif) ligand 1) expression, a chemokine attractant that inhibits infiltration of immune-inflammatory cells into the tissue microenvironment and subsequently represses anti-tumor immunity. They also found that Mincle signaling, which is induced by necroptosis, stimulates adaptive immunosuppression in the tissue microenvironment by infiltrating myeloid cells. In addition, pro-inflammatory cytokines, IL-1α released by necroptotic cells, can also recruit inflammatory cells and incur inflammation, which can stimulate the proliferation of tumor cells and potentially facilitate neoplastic progression. Further, the release of DAMPs (i.e., HMGB1 protein and ATP) from necroptotic cells into the tissue microenvironment can directly trigger inflammation. Najjar and colleagues [52] found that activation of RIPK1 and 3 kinase activity in an LPS-induced macrophage promotes cell death-independent inflammation by constant activation of c-Fos, Erk, and NF-κß, which are essential for the release of inflammatory cytokines. Additionally, necroptosis-induced inflammation can also release ROS and reactive-nitrogen intermediates (RNI), which are associated with DNA lesions and lead to genomic instability, thereby facilitating tumorigenesis. For instance, under chronic inflammation, activated macrophages produce and secret ROS/RNI and cytokines, such as TNF-α, which further enhances ROS generation through Rac-cytosolic phospholipase A2-leukotriene B4 cascades [190]. Further, necroptosis itself can also directly trigger ROS generation. For example, Zhang et al. [14] described that activated RIPK3 kinase increased ROS levels in NIH3T3 cells through an enhanced energy metabolism process. 

Finally, various experimental evidence has observed the ability of necroptosis in cancer metastasis. Extravasation is the process in which cancer cells exit from the blood vessels and enter into distinct sites, which is a very critical step in cancer metastasis. Strilic et al. [191] showed that human and murine cancer cells can induce necroptosis in endothelial cells and thereby stimulate cancer cell extravasation and metastasis through death receptor 6 (DR6) activation. Moreover, this study also found that binding of DR6 to its ligand amyloid precursor protein (APP) led to necroptotic endothelial cell death and tumor cells metastasis and extravasation. Ando et al. [192] reported that necroptotic pancreatic cancer cells showed higher migration and invasive capacity by the release of CXCL5 and CXCR2. 

## 5. Therapeutic Applications of Necroptosis Regulation

**Figure 5:**
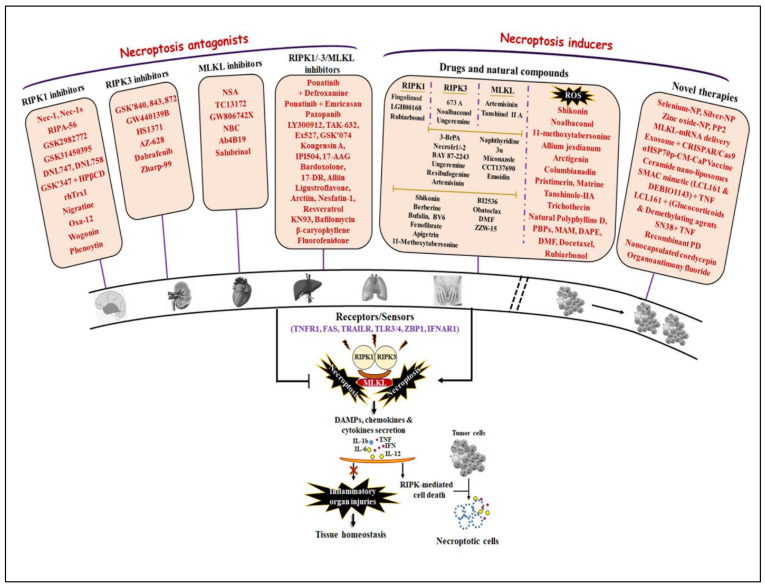
Classification of necroptosis-targeting therapeutic strategies in human clinical diseases.

In the past decades, much research has been conducted since the discovery of the first necroptosis inhibitor Nec-1, a specific target of RIPK1 [9,193]. Understanding necroptotic signaling has been growing, which is accompanied by findings of novel classes of necroptosis-targeting compounds. As necroptosis is not only involved in the maintenance of organismal homeostasis, but also forms etiological determinants of many human pathologies, at least two therapeutic models may be proposed: (i) activation of necroptosis to bypass accrued apoptosis- and drug-resistance in most cancer cells; (ii) inhibition of necroptosis to limit the loss of post-mitotic cells in inflammatory, toxic syndrome, and I/R injury-mediated pathologies. In this part, we reviewed a panel of a novel class of drugs that specifically targets necroptosis either via modulating expression or the activity of direct/indirect mediators of necroptosis. Some of them were successfully tested in preclinical and clinical trials (Figure 5). 

### 5.1. RIPK1 Antagonists

Necrostatins (Necs) are small tryptophan-like compounds that specifically inhibit RIPK1 kinase activity [193]. In recent years, the protective and anti-inflammatory effects of Nec-1 are identified in several types of human pathological diseases that efficiently alleviate the progression of the disease or improve prognosis. Nec-1 and its analogs are regarded as potential necroptotic inhibitors, although they have also off-target effects, moderate stability, and poor pharmacokinetic properties [194]. RIPA-56 (N-benzyl-N-hydroxy-2,2-dimethylbutanamide), a type-III kinase inhibitor, has been identified to inhibit RIPK1 and had shown a protective effect on mice against TNF-mediated mortality and multiorgan failure in SIRS diseases [195]. In addition, two other RIPK1 antagonists such as GSK2982772 and GSK31450395 have been developed by GlaxoSmithKline (GSK); among them, GSK2982772 is currently being evaluated in phase IIa trials for rheumatoid arthritis (NCT02858492), ulcerative colitis (NCT02903966), and psoriasis (NCT02776033) [196,197], and GSK31450395 was withdrawn from phase I clinical trials because no negative effects were observed on solid tumors growth and metastasis [198]. Moreover, other types of RIPK1 inhibitors DNL747 and DNL758 were developed by Sanofi and Denali, of which DNL747 is a blood–brain barrier (BBB) penetrable drug and has entered into clinical trials for the treatment of AD (NCT03757325) and ALS (NCT03757351), whereas DNL758 is unable to penetrate the BBB and is therefore used in the treatment for SIRS-associated diseases [199]. Yoshikawa et al. [200] discovered 7-oxo-2,4,5,7-tetrahydro-6H-pyrazolo [3,4-c]pyridine, a novel class of RIPK1 inhibitors with nanomolar potency as well as BBB permeability, which efficiently mitigated disease progression in an EAE mice model of MS. GSK’347 (RIPK1 inhibitor) in combination with 2-hydroxypropyl-β-cyclodextrin (HPβCD) showed efficient protection against Niemann–Pick disease in both animal models and human patients [201]. In addition, another new RIPK1 inhibitor recombinant human Trx-1 (rhTrx-1) has been found to improve neurological deficits in an I/R-MCAO mice model through suppressing RIPK1 expression [202].

### 5.2. RIPK3 Antagonists

Apart from the RIPK1 inhibitors, GSK also developed some small molecules of RIPK3 antagonists, GSK’840, 843, and 872, that can inhibit necroptosis. However, therapeutic properties of these inhibitors are undermined due to their failure to inhibit TNF-α and TRAIL-induced apoptosis [203,204]. Dabrafenib, originally discovered as a B-rafV600E inhibitor, has also been identified to inhibit RIPK3 kinase activity in vitro, and it rescued hepatic cells from necroptotic cell death, but with little effect on apoptosis [166]. Moreover, Dabrafenib treatment attenuated neurological deficits in the I/R brain injury mice model by the suppression of RIPK3-dependent necroptosis [205]. GW440139B, identified through screening a library of 8904 bioactive compounds, was identified as a potent RIPK3 inhibitor that seems to obstruct RIPK3-dependent MLKL phosphorylation and successively inhibits necroptosis [206]. Additionally, Park et al. [207] discovered a novel class of necroptosis inhibitor HS1371, which was observed to inhibit RIPK3 kinase activity in vitro in an ATP-competitive manner. Recently, through in silico binding assay and the CMap approach AZ-628, a potent RIPK3 antagonist was discovered, which seemed to modulate TRIM24-RIPK3 axis and targeted RIPK3-kinase activity, resulting in reduced osteoarthritis pathogenesis in the DMM mice model [208].

### 5.3. RIPK1/-3 Antagonists

Moreover, recently, a novel type of RIPK1/3 dual antagonist has been identified, which efficiently inhibits necroptosis in human and murine cells. For instance, Pazopanib and Ponatinib, receptor tyrosine kinase inhibitors with anti-tumor activity, were identified to specifically inhibit necroptosis, but not apoptosis, induced by multi-ligand receptors [209]. Both inhibitors specifically blocked RIPK1 and RIPK3 kinase activities through competitively binding to the ATP pocket of the enzyme. Ponatinib diminished RIPK1, RIPK3, and MLKL phosphorylation and preferentially inhibited interactions between RIPK3 and MLKL. Pazopanib specifically targeted RIPK1 and had no impact on the interaction between RIPK3 and MLKL. These two compounds efficiently blocked necroptosis; however, their therapeutic values are not promising, as they started showing cardiotoxic effects. However, a combination of Ponatinib with deferoxamine attenuates myocardial injury in both in vitro and in vivo cardiac injury models by simultaneous suppression of necroptosis and ferroptosis [210]. Similarly, in another study, Ponatinib in combination with emricasan significantly alleviated neuronal injury in an I/R-MCAO brain injury rat model via inhibiting RIPK1/-3/MLKL phosphorylation [210]. In addition to Ponatinib therapy, an organosulfur compound alliin alone exerted the cardioprotective effects in an in vitro and in vivo myocardial infarction model via reducing necroptosis related RIPK1, -3 and TNFR1/2 proteins expression and, escalating the autophagy machinery [211]. Furthermore, a pan-RAF inhibitor LY300912 was identified to block necroptosis through declined phosphorylation of RIPK1/-3/MLKL and thus may represent a promising therapeutic drug for treatment of colitis [212]. Recently, another pan-RAF inhibitor TAK-632 was identified to directly inhibit RIPK1 and RIPK3 kinase activity and showed effective protection to human and murine cells against TNF-α mediated necroptosis rather than apoptosis, including inflammation in SIRS disease [37]. Ex-527, a potent inhibitor of Sirt1 deacetylase, efficiently improved I/R brain injury by suppressing RIPK3 and MLKL expression [213] and passed phase IIa clinical trials for Huntington’s disease [12]. Likewise, pretreatment with bafilomycin-A1, a late-stage inhibitor of autophagy, significantly attenuated brain infarct volume and neurological deficits in a global I/R cerebral injury rat model via inhibiting autophagy-triggered necroptosis, as evidenced by reduced RIPK1/-3 proteins level as well as GLUD1 activity [214]. In addition, pretreatment of CaMKs inhibitor KN-93 efficiently ameliorated tGCI-induced neuronal injury in the rat hippocampal region by blocking RIPK1-3 interaction and Drp1-mediated mitochondrial fission [215]. Recently, another novel compound of necroptosis inhibitor, Oxa12, was identified that shows an efficient protection to MPTP PD mice model from zVAD-fmk-induced necroptosis and dopaminergic neuron loss in PD pathogenesis [216].

### 5.4. MLKL Antagonists

Over the last years, various MLKL inhibitors have also been developed that were efficiently involved in the control of necroptosis. Necrosulfonamide (NSA), which specifically targets MLKL, is bound to a Cysteine-86 (Cys-86) of human MLKL and creates a covalent adduct to inhibit MLKL oligomerization, resulting in inhibition of necroptosis. Recently, Jiao et al. [202] found that NSA treatment alleviated neurological dysfunctions in spinal cord neuronal injury (SCI)-mice and OGD-mediated SCI by blocking MLKL activation, but independently of RIPK3 phosphorylation and thus may open a therapeutic window for SCI treatment. Furthermore, GW806742X was also identified to directly bind to the MLKL pseudokinase domain in an ATP-dependent manner and successively inhibit necroptosis through blocking phosphorylation of MLKL, oligomerization, and plasma membrane translocation [217,218]. TC13172, a novel class of MLKL inhibitor with nanomolar potency, was found to specifically bind to MLKL at Cys-86 site and can consequently block plasma membrane translocation rather than phosphorylation [217]. In recent studies, two other MLKL inhibitors were also identified, Ab4B19 (TrkB agonist antibody) and salubrinal (ER stress inhibitor), which were found to exert neuroprotective effects in an I/R cerebral injury mice model through suppressing the levels of MLKL and p-MLKL [219,220].

### 5.5. Hsps 90/70s Inhibitors

Several antagonists of Hsp90, a molecular chaperone that is involved in the stabilization and functions of a principal necroptotic mediator (RIPK3 and MLKL), such as kongensin A [221], IPI-504, 17AAG [222,223], 17-demethoxy-reblastatin [224] and alvespimycin (17-DMAG) [225], have been documented to impair necroptosis. Therefore, these inhibitors interfering with Hsp90 function might be a new therapeutic approach for the treatment of necroptosis-related human pathologies [226]. Further, IPI-504 and 17AAG have completed phase IIa clinical trials in human patients with different types of cancer [227,228]. In addition, Wang et al. [229] discovered a novel orally active necroptosis antagonist bardoxolone (CDDO 7) that seems to target Hsp90, leading to diminished RIPK1 and RIPK3 phosphorylation and providing efficient protection against TNF-α-induced necroptosis in mice models of both SIRS and cerebral I/R injury. Apart from the above Hsp90 inhibitors, other small molecules, namely necroptosis-blocking compound 1 (NBC1) and JG-98, were identified, which seemed to inhibit allosteric transition in Hsp70 by covalently binding, due to which Hsp70 loses the capability to promote MLKL polymerization, and which finally caused necroptosis inhibition in human ND and cancer [27,230].

### 5.6. Cancer Antagonists

Various natural compounds have also been documented that function as necroptosis modulators. Pretreatment with traditional Chinese medicine extracts such as ligustroflavone and β-caryophyllene efficiently improved neurological deficits in the I/R MCAO mice model by downregulating RIPK1/-3/MLKL expression [212,231]. Moreover, the clinically used anti-convulsant, phenytoin, as well as natural compounds found in different plants (wogonin and fluorofenidone), were found to ameliorate renal injuries in cisplatin- and IRI-induced AKI mice models by inhibiting RIPK1 kinase activity and blocking RIPK1/-3/MLKL phosphorylation [232,233,234]. Additionally, some other natural compounds such as arctiin [235], nesfatin-1 [236], resveratrol [237], and Tanshinone-I [238] have also been documented to show cardioprotective effects in H/R-treated H9c2 cell lines and I/R SD rats via inhibiting RIPK1/-3/MLKL phosphorylation. Further, a growing number of cancer studies also found the role of some natural compounds in necroptosis induction in cancer cells, wherein many types of cancer cells have defective apoptosis induction or have acquired apoptosis resistance. For instance, Shikonin is the first natural product found to trigger necroptosis in various types of cancer cells, including pancreatic cancer [239], glioma [240], myeloma [241], osteosarcoma [242], nasopharyngeal carcinoma [243,244], lung cancer [245] and triple-negative breast cancer cells [246]. Furthermore, for RIPK1/3 activation and necrosome complex formation, shikonin also provoked oxidative stress in glioma, gastric, nasopharyngeal, and breast cancer cells, which inhibits growth and metastasis of cancer cells through necroptosis induction. These observations suggest that oxidative stress is a regulative factor in shikonin-induced necroptosis. It has also been reported that treatment of shikonin overcomes drug resistance in drug-sensitive MCF-7 and HEK293 cancer cell lines via induction of necroptosis. Noalbaconol, a novel small-molecular compound isolated from *Albatrellus confluens*, has been found to induce necroptosis in some kinds of cancer cells [247,248]. In-depth, noalbaconol triggered necroptosis through promoting downregulation of E3-ubiquitin ligases, proteasomal degradation of cIAP1/2 and TRAFs protein to prevent RIPK1 ubiquitination caused by RIPK3-mediated ROS generation. The increased levels of ROS are also a regulative factor in several other types of naturally derived necroptosis inducers such as 2-methoxy-6-acetyl-7-methyljuglone [249,250,251], 11-methoxytabersonine (11-MT) [252], *Allium jesdianum* [253], Arctigenin [254], Columbianadin [255], Matrine [256], Natural Polyphyllins D [257], Pristimerin [258] and Trichothecin [259], thus further confirming their anti-carcinogenic role via necroptosis induction. Berberine induces necroptosis in ovarian cancer cells and MYC-overexpressing lymphomas by promoting mitophagy-mediated RIPK1/3 activation and necrosome complex formation [260,261]. Bufalin triggered necroptotic cell death in glioma by promoting necrosome complex formation upon caspase-8 inhibition [262]. Emodin, an anthraquinone derivative, has been reported to increase TNF-α, RIPK1, and RIPK3 levels in in vitro and in vivo glioma cancer models, which significantly inhibited glioma progression through necroptosis induction [263]. Ungeremine, an alkaloid derivative, has been shown to overcome drug resistance in many cancer cells through escalating RIPK3-mediated necroptosis [264]. Resibufogenine, a naturally occurring bioactive compound extracted from toad venom, has been shown to inhibit the growth and metastasis of colon cancer via triggering RIPK3/MLKL-dependent necroptosis in both in vitro and in vivo colon cancer models [265]. Ophiopogonin D, a natural triterpenoid saponins compound, has been shown to induce RIPK1-mediated necroptosis in androgen- and prostate-specific antigen-dependent LNCaP prostate cancer cells [266]. 11-Methoxytabersonine, an aspidosperma-monoterpenoid indole alkaloid, also possessed antitumor activity in A549 and H157 lung cancer cell lines via the induction of necroptosis with autophagy through the activation of AMPK/mTOR and JNK signaling pathways [252]. Ganoderic acid T (GAT), a natural triterpenoid extracted from the *Ganoderma lucidum*, has been found to sensitize HeLa cancer cells to gamma-ray radiotherapy via switching from apoptosis to necroptotic cell death [267]. Delehouzé et al. [268] discovered that Nigratine, a natural flavonoid, specifically targets RIPK1-kinase via a non-ATP competitive manner, which seems to inhibit both necroptosis and ferroptosis and thus is capable of protecting human aortic endothelial cells and porcine renal cells from cold hypoxia/reoxygenation-mediated cell death. Rubiarbonol-B (Ru-B), a natural arborinane triterpenoid extracted from *Rubia philippinesis*, switches apoptosis to necroptosis by upregulation of RIPK1 phosphorylation and NOX-1-derived ROS generation in colorectal cancer cells [269]. Bhosale et al. [270] reported that administration of apigetrin, a glycoside dietary bioactive flavonoid, triggered both apoptosis and necroptosis in human Hep3B hepatocellular cancer cells via inhibition of NF-κB subunit phosphorylation (p-p65) and the IκB pathway. In addition, protein-bound polysaccharides (PBPs) have shown anti-tumor activity in MCF-7 breast cancer cells and SKMel-188 melanoma by ROS-mediated necroptosis [271]. However, RIPK3 is not universally expressed in many cancer cells (Koo et al., 2015); thus, MLKL, a final executioner of necroptosis signaling pathway, could be a potential target in these type of cancer cells. Liu et al. [272] identified tanshinole II-A, a natural diterpenoid, which was found to specifically trigger MLKL-mediated necroptosis through increased ROS levels in A549 lung cancer cells.

Furthermore, several necroptosis-targeted reagents have also been found in different types of cancer treatment research. BV6, a small SMAC mimetic molecule, was reported to sensitize acute myeloid leukemia cells to cytarabine-mediated cell death and antagonize IAP proteins to circumvent apoptotic resistance through necroptosis induction under caspases inhibition (not by zVAD-FMK), which is further significantly suppressed by Nec-1 or NSA [273]. Moreover, combinational treatment with TNF-α showed synergistic anti-tumor effects in pancreatic cancer cells [274]. Cabal-Hierro et al. [275] showed that monotherapy with a topoisomerase inhibitor SN38 (an active metabolite of irinotecan), or combined with TNF, induces cytotoxic effects through activation of TNF/TNFR signaling and RIPK1-dependent necroptosis in colon cancer cells and inhibits tumor growth progression in colon tumors of xenografted female SCID mice. This indicates its potential use in TNF-based signaling reactivation to increase its therapeutic efficiency in colorectal cancer. 1,2-Diarachidonoyl-sn-glycero-3-phosphoethanolamine (DAPE) has been shown to induce necroptotic cell death in NCI-H28 pleural mesothelioma cells through enhanced RIPK1/-3-mediated ROS levels [276]. 3-Bromopyruvate (3-BrPA), an alkylating agent, triggers necroptosis by upregulating the RIPK3/MLKL/DRP1 axis independently of RIPK1 activation [277]. In addition, Guan et al. [278] found that treatment of NecroIr1 and -2 complexes eradicated drug-resistant cancer cells by the induction of necroptosis events, including unregulated extracellular Ca^2+^ efflux, leakage of LDH and HMGB1, higher ROS generation, loss of ∆Ψm, and lastly, activation of RIPK3/MLKL-dependent necroptosis. Sulkshane et al. [279] demonstrated that Obatoclax (GX15-070), a Bcl-2 antagonist, promoted necroptosis in in vitro and in vivo oral cancer models and in rhabdomyosarcoma cells via recruiting the RIPK1/-3/MLKL complex onto the autophagosome membrane, resulting in tumor growth suppression in these cancer models. In another study, pretreatment with BAY 87-2243, a specific inhibitor of mitochondria complex-I, triggered ROS generation, mitochondrial dysfunction and mitophagy, following RIPK3/MLKL activation and induction of necroptosis in BRAFV600E melanoma cells. Fingolimod (FYT720), a sphingolipid analog drug for MS, has been found to directly bind and inhibit sI2PP2A/SET oncogene, leading to activation of PP2A, a tumor suppressor enzyme, which further upregulates RIPK1-dependent necroptosis to constrain cell growth in A549 human lung cancer cells and in SCID mice implanted with A549/sh-I2PP2A/SET xenografts [280,281]. Zhang et al. [282] found that FYT720 triggered necroptotic cell death in both in vitro and in vivo models of glioblastoma by activating RIPK1/3-dependent ROS-JNK-p53 axis. Dimethyl fumarate (DMF), an FDA-approved immunomodulatory drug for MS, has been reported to induce necroptotic cell death in CT26 colon cancer cells via less glutathione content, ROS overgeneration, mitochondrial dysfunction, and MAPKs activation [283]. Docetaxel, a taxane-derived anti-tumor drug, sensitizes MDA-MB-231 triple-negative breast cancer cells and breast cancer tumor xenograft nude mice to cell death through ROS-mediated BAD-induced necroptosis [284]. Methyl methanesulfonate (MMS), an alkylating agent, was reported to induce necroptotic cell death in A549 human lung cancer cells, possibly by upregulating the PIG-3-ROS signaling pathway [285]. ZZW-115, a trifluoperazine derivative, showed concentration-dependent tumor inhibition via directly targeting NUPR1 and necroptotic cell death in pancreatic cancer cells [286]. Kong et al. [287] identified a novel naphthyridine derivative 3u that activated RIPK3/MLKL-dependent necroptosis at lower concentrations while it induced apoptosis at higher concentrations in A375 human melanoma cells. The antifungal drug miconazole was reported to induce both apoptotic and necroptotic cell death in human MDA-MB-231 breast cancer cells via augmented Bax/Bcl-2 ratio, upregulation of RIPK3 and MLKL phosphorylation levels, and ROS generation [288]. Fenofibrate is an FDA-approved standard drug that is widely used to reduce the high triglyceride and high cholesterol levels in the blood. You et al. [289] found that cytotoxic activity of fenofibrate on human hep3B lung cancer cells was induced by inhibition of lipid-metabolism gene expression concurrently with both apoptosis and necroptosis induction. Further, a novel CHOP activator LGH00168 exhibited anti-tumor activity in A549 cells and mice bearing lung tumor xenografts via ROS-mediated loss of ∆ψM, ER stress, NF-κβ inhibition, and RIPK1-dependent necroptosis [290]. CCT137690, an aurora kinase inhibitor, has been found to indirectly induce RIPK3/MLKL-dependent necroptosis, which further leads to inhibition of pancreatic carcinoma progression [291]. Chefetz et al. [292] identified a Pan-ALDH1A antagonist, 673A, which was found to alleviate chemoresistance and stemness in ovarian cancer stem-like cells by triggering RIPK3-dependent necroptosis. Li et al. [293] found that PP2, a well-known SRC inhibitor, disrupts RIPK3 oligomerization function, resulting in inhibition of phosphorylation and oligomerization of MLKL, which is suggested as a promising therapeutic strategy for Crohn’s disease development. Recently, organoantimony (III) fluoride has also been shown to induce RIPK3-MLKL-dependent necroptosis through significant GSH depletion and ROS elevation in human MDA-MB-231 breast cancer cells [294].

With advances in technology, recent studies have developed a novel anti-tumor therapy based on natural delivery platforms for anti-cancer therapeutics such as metal nanoparticles, non-coding RNAs, engineered exosomes, and oncolytic viruses with greater target efficacy and minimal side effects and thus are seen as promising therapeutic strategies for the treatment of cancer. Sonkusre et al. [295] showed that the combination of selenium (Se), an essential trace element in the human body, with nanoparticles induces necroptotic cell death by the upregulation of death receptors, mainly TNF-α, and excessive ROS generation in prostate adenocarcinoma (PC-3) human cell lines. Zinc oxide nanoparticles (ZnO-NP) significantly induce necroptosis by the upregulation of RIPK1, RIPK3, and MLKL expression in human MCF-7 breast cancer cells [296]. Recently, Kaokaen et al. [297] identified that administration of nanocapsulated cordycepin (CS) with high concentration switches from apoptotic to necroptotic cell death through a reduction in apoptotic gene expression and higher ROS-mediated inhibition of autophagy in human oral cancer cells (HSC-4). Furthermore, intratumor MLKL-mRNA delivery synergizes with immune-checkpoint blockades (ICBs), impedes lymphoma growth in mice, and improves anti-tumor activity in syngeneic mice with colon carcinoma and melanomas [298]. In addition, delivery of engineered self-tumor-derived exosomes with CRISPR/Cas9 vectors triggered necroptosis in cancer cells via activation of TNF-α receptor signaling pathways together with impairment of caspase-8 and IAP1/2 genes expression, resulting in the induction of necroptotic cell death concurrent with the production of anti-tumor immunity against cancer cells [299]. In B16OVA mice melanoma models, combining nano-size vaccines composed of “biomimetic artificial necroptotic cancer cells” (αHSP70p-CM-CaP) with anti-PD1 antibodies completely blockades cancer progression and activates the immune system against cancer cells for the long term [300]. In another approach, the intra-tumoral injection of necroptotic fibroblasts combined with ICBs led to increased tumor progression-free survival rates and provided long-term activation of anti-cancer immune response [301]. Zhang et al. [302] found that administration of ceramide nano-liposomes (CNLs) via a nano-scale delivery platform significantly induced selective cell death by promoting MLKL-dependent necroptosis in A2780cp, SKOV3, and PE04 cisplatin-resistance ovarian cancer cells and in ovarian tumor cells of xenografted mice. In the cholangiocarcinoma model, treatment of both SMAC-mimetic (LCL161 and DEBIO1143) and TNF sensitizes cancer cells to a low dose of gemcitabine-induced necrosome complex formation and necroptosis upon the loss of cIAPs and caspase-8 function [303]. SMAC-mimetics together with some demethylating agents (i.e., 5-azacytidine (5AC) and 5-aza-2’-deoxycytidine (DAC)) or glucocorticoids were reported to induce necroptosis in apoptosis-resistant acute lymphoblast leukemia cells [304,305]. In a recent study, short-term pharmacological inhibition of ADAM17 via delivery of recombinant prodomain (PD) impairs TNF-induced necroptosis in tumor endothelial cells and subsequently blocks metastasis [306]. 

Such a role of natural compounds and drugs in regulating necroptosis will hopefully provide prospective approaches to manipulate tumor cell death. However, further studies are warranted to examine the efficacy and the possible harmful effects of these necroptosis-targeting compounds for antitumor chemotherapy; further therapeutics can be combined with selective necroptosis inducers to cancer-specific agents or antibodies.

## 6. Conclusions and Future Perspectives

While studies on cell death mechanisms for several years have focused mainly on apoptosis, in recent years, necroptosis has become increasingly attractive in this field, given its implications in human pathologies, including cancer, providing clear evidence that both necroptosis and apoptosis are major modes of programmed cell death. The molecular signaling of necroptosis is a complex process, is tightly interconnected with multiple regulatory pathways, and contributes in the basic physiological functions such as immune response, inflammation, embryonic development, and the maintenance of tissue homeostasis. Furthermore, the specific regulation of major necroptotic regulators such as RIPK1, RIPK3, and MLKL is crucially involved in the selection process, wherein cells survive or die from apoptosis or necroptosis. The evidence of necroptosis’ impact on processes and systems was considered completely unrelated; however, not long ago, its impact accumulated rapidly, and we have no doubt that this research area contains countless exciting discoveries waiting to be revealed.

Targeting necroptosis signaling pathways may be the most promising therapeutic way to treat various diseases. Through further investigations in such areas, we will be more aware of necroptotic cell death programming, and we can develop new opportunities regarding the exploration of the pathogenesis of many pathologies. Due to its critical role in organ growth and cancer cell proliferation, targeting RIPK1/RIPK3/MLKL might be a key promising therapeutic approach for multiple severe pathologies. Thus, by using small molecules of necroptosis inhibitors, it may be possible to improve severe disease symptoms and prolong the survival of patients. Exploiting necroptotic cell death pathways may also give us new opportunities to target specific cancer subpopulations, such as cancer stem cells that are poorly affected through accrued apoptotic and drug resistance. Therefore, in the coming years, further investigation in this area may include the discovery of novel classes of RIPK1, RIPK3, and MLKL inhibitors. As a key executioner of necroptotic cell death, MLKL appears to be the leading mediator of necroptosis. Therefore, in the future, studies exploiting novel types of MLKL inhibitors may be a crucial step for the treatment of necroptosis-associated diseases. Additionally, the search for novel therapeutic strategies with off-target effects that are capable of specific and thoroughly controlled induction of necroptosis in human pathologies, including tumor cells, gives first priority aim for future research in the field of necroptotic cell death.

## Figures and Tables

**Table 1:** Key markers of programmed cell death signaling used to differentiate between apoptosis and necroptosis.

	Apoptosis	Necroptosis	References
Morphology	Cytoplasmic shrinkage, Chromatin condensation, DNA fragmentation, Cell membrane blebbing, Apoptotic body formation and shedding	Translucent cytoplasm, Swelling sub-cellular membrane organelles, Increased cell volume, Early loss of membrane integrity, Spillage of intracellular contents into extracellular space	[1,3,5,6,9]
Death signaling factors	BID, BAX, Bcl-2, Cytochrome c, APAF1, FADD, Caspase-8, Caspase-9	RIPK1, RIPK3, FADD, Caspase-8, cFLIPs, cIAPs, CYLD	[1,3,9,14,23]
Death execution factors	Caspase-3, Caspase-7	p-MLKL (core executioner), PGAM5, Drp1	[24,25,26,27]
Death execution events	Caspase-3 activation after interacts with caspase-8 and -9 caused cell shrinkage, DNA fragmentation, shedding of apoptotic body formation	MLKL phosphorylation, oligomerization and plasma membrane translocation causes membrane permeabilization and necroptosis induction	[3,24,28]
Methods to examine death events	TUNEL assays, DNA laddering, Annexin-V/PI positive cells counting, Caspase-3/-7 activity assays	PI staining (staining with impermeant dyes), HMGB1 & LDH release assay, Detection of RIPK1, RIPK3 and MLKL expression and membrane localization, Detection of necroptosis cells morphology	[24,25,26,27,29]

**Table 2:** Role of necroptosis in human pathological diseases.

No.	Pathological Diseases		Disease Models	Involvement of Necroptosis	References
1	**Neurological disease**	Multiple sclerosis	Postmortem brain tissue samples of human patients; RIPK3^−/−^ and Cuprizone-induced MS mice model	Increased RIPK1/-3 expression in microglia and macrophages in both human and mouse brain samples,Reduced cleaved caspase-8 expression	[69,70]
			EAE mice model	NF-κβ signaling mediated inflammatory response promotes FLIPL expression leading to necroptosis without caspase-8 activation	[70,71,72]
				Elevated expression of RIPK1, increased RIPK3 and MLKL phosphorylation in both microglia and oligodendrocytes primarily localized in the white matter of brain.	[70,73]
		Amyotrophic sclerosis	*SOD1^G93A^*-mutated mice	Increased levels of RIPK1 and RIPK3 in MNs and astrocytes primarily localized in the spinal cord	[74,75,76]
			*Optn*^−/−^ mice	Elevated levels of RIPK1, RIPK3 and pMLKL and necroptosis in the MNs of spinal cord	[76]
			*TBK1*^−/−^ mice; *TBK1*^−/−^ cell line	Higher level of RIPK1 and TNF-α induced necroptosis	[77,78]
		Alzheimer’s disease	Postmortem brain tissue samples and 23 AD, 24 p-preAD brain tissue samples from human patients; 5xFAD mice model	Elevated level of RIPK1/-3/MLKL at both mRNA and protein level	[79,80,81]
			Tg2576 APP/PS1 mice model	Higher RIPK1 activation drives Cst7 expression in microglia leads to lysosomal dysfunction and blocked proteostasis of deposit Aβ-plaques in AD	[80,82]
			*cpdm* mice model	Attenuation of M1 ubiquitination of RIPK1 to stimulate its activation	[83]
		Parkinson’s disease	OPA1-mutant PD patients derived iPSC; OPA1-A495V and OPA1-G488R neuronal cell lines	Higher phosphorylation level of RIPK1/-3/MLKL and mitochondrial dysfunction-induced necroptosis	[84]
			PD models (PC12 & SH-SY5Y cell line; 6-OHDA & MPTP mice model)	Promotes necrosome complex formation (RIPK1/-3/MLKL),ROS stimulated RIPK1 activation following with ASK1/JNK signaling activation	[84,85,86]
2	**Renal disease**	I/R-induced AKI	IRI C57BL/6 mouse model	RIPK3- and CypD and PPIF mediated mPT-dependent necroptosis	[87,88,89]
		Toxicant-induced AKI	FA-AKI renal mouse model	RIPK3/MLKL- and TWEAK-Fn14 axis-induced necroinflammation mediated necroptosis	[90,91]
			Cisplatin & Oxalate crystals nephrotoxicant mice model	RIPK3- and CypD-induced necroinflammation mediated necroptosis in the renal proximal tubular epithelial cells and whole kidney lysates,MLKL dependent necroptosis via higher MLKL membrane association in oxalate crystal-treated renal PTC cells	[92,93,94,95]
		Sepsis-induced AKI	CLP C57BL/6N mice model	RIPK3 induced higher OS and mitochondrial dysfunction via upregulation of NOX4 and mitochondrial complex-I/III inhibition	[96]
		Renal fibrosis CKD	RIPK3^−/−^ UUO and AD fibrosis mice model	RIPK3 exacerbates kidney fibrogenesis via the PI3K-AKT dependent activation of ACL metabolic activity	[95]
			IRI mice model; OGBD treated PTC cell line	RIPK3/MLKL-dependent necroptosis mediated necroinflammation promotes renal interstitial fibrogenesis	[97]
3	**Hepatic disease**	Alcoholic liver disease	Chronic alcoholic mice model	CYP2E1-mediated higher RIP3 expression induces hepatocyte necroptosis during chronic alcohol treatment	[98]
			Gao-binge (chronic alcoholic) mice model	Impaired hepatic proteasome function via chronic alcohol exposure led to the higher accumulation of RIPK3 and induced necroptosis	[99,100]
		NAFLD and NASH diseases	HFCD and MCD diet mice model	TNF-α mediated RIPK3-dependent necroptosis, -oxidative stress and -necroinflammation in mice liver lysates	[101,102,103,104]
			FFC diet mice model	MLKL-dependent necroptotic signaling promotes FFC diet-induced liver injuries and necroinflammation via autophagy-flux inhibition	[105]
4	**Pulmonary disease**	Acute lung injuries (ALI)	VILI- induced ALI mice model	Impaired mitochondria-FAO metabolism pathway via MV exposure led to the RIPK3-dependent necroptosis mediates ALI pathogenesis	[106]
			LPS/ATP-induced ALI mice model	RIPK3 mediated NLRP3 inflammasome activation promotes lung injury via markedly increase inflammatory cell influx,IL-1α/β, IL-6, IL-18 secretion and HMGB1 release in infiltrating macrophages/monocytes cells	133
		Acute respiratory distress syndrome	Oleic acid-fed SD rat ARDS model	Elevated expression of TNF-α and RIPK1, RIPK3, MLKL in the BALF and lung tissue of OA-fed SD rat	[107]
			LPS-induced ARDS mice model	RIPK3-dependent necroptosis enhanced necroinflammation, ROS overgeneration and lung tissue injuries via increasing neutrophil infiltration and cytokines secretion in lung parenchyma tissue	[108,109]
		Sepsis/systemic inflammatory associated disorder (SIRS)	TNF-α induced SIRS mice model	RIPK1/-3 induced necroptosis with the DAMPs release and pro-inflammatory cytokines secretion associated with higher systemic inflammation and polymicrobial sepsis	[109]
			Cecal slurry-induced neonatal sepsis C57BL/6 mouse model	Elevated expression of RIPK1 and RIPK3 associated with systemic and pulmonary inflammation and lung injuries in neonatal sepsis	[110,111]
		Chronic obstructive pulmonary disease	Cigarette smoke (CS)-exposed Beas-2B cells and - C57BL/6 mice model	CS-induced mitoROS, mitochondrial dysfunction and mitophagy leads to necroptotic signaling activation with necroinflammation in pulmonary epithelial cells and alveolar macrophages	[112,113,114,115,116]
		Idiopathic pulmonary fibrosis (IPF)	BLM-induced IPF mice model	RIPK3 mediated necroptosis and DAMPs release induced necroinflammation and lung injury via increasing neutrophil infiltration and cytokines secretion in the alveolar epithelial cells	[117]
5	**Cardiovascular disease**	Heart failure	I/R or cardiotoxicant (doxorubicin) induced cardiac injury murine and pig model	Elevated level of RIPK1, RIPK3 and MLKL (phosphorylation) in the rat myocardial tissues, Detection of RIPK1/RIPK3 necrosome complex formation,Higher RIPK3 level caused activation of CAMKII as a results MPT opening and myocardial necroptosis	[118,119,120,121,122]
		Atherosclerosis	OxLDL- or APOE-deficient mice model	Higher RIPK3 activation and subsequent necroptosis induction atheroma macrophages leads to the local and systemic inflammation and atherosclerotic lesions	[123,124]
		Aortic aneurysm	Elastase-induced AAA C57BL/6 mice model	Increased RIPK1 and RIPK3 level in aneurysmal tissues promotes AAA progression via caused SMC necroptosis and vascular inflammation (lymphocytes infiltration and DAMPs release)	[125,126]
			Calcium phosphate-induced AAA C57BL/6 mice model	GSK'074 treatment diminished AAA progression by impeding SMC necroptosis, necroinflammation (macrophages infiltration and cytokines secretion) and aortic expansion in aneurysm-prone aortae	[127]
6	**Autoimmune diseases**	Psoriasis	Primary epidermal keratinocytes cultured cells; Aldara-/IMQ- induced psoriasis mice models	Enhanced RIPK3 expression facilitates psoriatic inflammation by neutrophil-mediated cytokines/chemokines secretion,Elevated RIPK1/-3/MLKL expression, detection of their localization in all layers of epidermal skin cells confirmed by confocal microscopy.	[128,129,130]
		Rheumatoid arthritis (RA)	Collagen-induced arthritis mice model	Enhanced RIPK1/RIPK3/MLKL expression promotes NLRP3 inflammasome complex formation and cytokines secretion in the synovium joint tissue,IFN-γ diminished necroptosis-mediated RA progression by downregulation of MLKL expression	[131,132]
7	**Cancer disease**	Acute myeloid leukemia (AML)	DA1-3b p210^BCR-ABL^ leukemia murine cell line; DA1-3b/C3HeOuJ mouse model	Reduced RIPK3 expression observed in clinical samples from human AML patients was independently associated with poor prognosis,RIPK3 downregulation suggest diminished necroptosis via p65/RelA cleavage and as results shift towards NF-κβ mediate cell survival in DA1-3b leukemia cells	[133]
		Chronic lymphocytic leukemia (CLL)	Primary CLL cells from human CLL patients	Lower expression of RIPK3 and CYLD reduced TNF-α/zVAD mediated necroptosis in CML cells	[134]
		Head and neck squamous cell carcinoma (HNSCC)	Primary and metastatic cell lines and tissues from human HNSCC patients	Enhanced genomic methylation of RIPK1 promoter caused reduced TLR3-mediated apoptosis/necroptosis with higher tumor cells migration in HNSCC metastatic cells,Loss of RIPK1 expression strongly linked with metastatic disease in a cohort of HNSCC patients	[135]
		Non-small cell lung cancer (NSCLC)	Clinical samples from 253 and 394 NSCLC human patients	Lower RIPK1/-3/MLKL expression was linked with poor prognosis in a NSCLC tissues associated with reduced levels of necroptosis signaling, Lower RIPK1 and PELI with higher p53 expression found to be linked with a worse OS of stage-I NSCLC patients	[136]
		Ovarian cancer	Clinical samples from 75 human ovarian cancer patients	Low MLKL level was significantly linked with both reduced DFS and OS of patients with ovarian cancer, MLKL serves as a potential prognosis marker in ovarian cancer patients	[137]
		Colon cancer	HT29, Colo205, HCT116, SW480 colon cancer cell lines; Colon cancer tissues samples from human colon cancer patients	Reduce RIPK1 and RIPK3 expressions (due to hypoxia) resists cancer cells to chemotherapeutic agents-induced necroptotic cell death; Poly I:C induces TLR3/RIP3-dependent necroptosis and tumor growth inhibition via induction of DC cells mediated anti-tumor activity in colon cancer tissues	[138]
		Cervical cancer	HPV18-positive HeLa and C4-I cervical carcinoma cell lines	RIPK3-upregulation stimulates IL1-α secretion from Poly I:C-induced necroptotic cervical cancer cells leads to activation DC cells, selective activation of DC cells shows anti-tumor effects via producing cytotoxic cytokine IL-12 or activating CD8^+^ T-cells	[139]
		Melanoma	DM733, DM598, DM738 and DM833 melanoma cell lines; A2058 cells injected NCr-*nu/nu* miceB16 mouse melanoma cell lines and B16 cells injected C57Bl/6 mice melanoma metastasis model	Selective inhibition of CYLD expression and activation of JNK/AP-1 and β1-integrin signaling by Snail is critical to protects melanoma from necroptosis in melanocytes and nevus cells, zVAD-fmk in combination with RT, CT, HT increased necroptosis in B16-F10 melanoma cells concomitantly with HMGB1 secretion and increase cytotoxic DCs as well as CD8^+^ T-cells infiltration around tumor microenvironment. RIPK3 upregulation induces NK T-cells mediated anti-tumor responses via mitochondrial PGAMF-NFAT/Drp1 signaling activation in metastatic melanoma tumor cells;Increased RIPK1 expression positively linked with the higher metastatic rate and a poor prognosis in melanoma potentially due to NF-κβ-mediated pro-tumorigenic abilities	[140,141,142]
		Glioblastoma	Tissue samples from human glioblastoma patients	Increased RIPK1 levels prevents p53 induction via augmenting mdm2 level and NF-κβ signaling activation to confers a poor prognosis in glioblastoma	[143]
		Lung cancer	HBEC-2, HBEC-13 and BEAS-2B cell lines; Cigarette smoke/Benzo(a)pyrene-exposed mouse lung tumor model	Higher RIPK1 expression promotes BPDE-induced malignant transformation of HBEC cells by catalase-mediated suppression of ROS overproduction as well as MAPKs signaling deactivation	[144]
		Breast cancer	Clinical samples of human breast cancer patients; MDA-MB231, 4T1, MCF10A and MCF12A breast cancer cell lines; MDA-MB-231/4T1 xenografted Balb/C mice model	Nearly 85% patients of breast cancer have reduced RIPK3 expression that was associated with a worse prognosis, Lower RIPK3 expression suggest by genomic methylation of transcription factor in its transcriptional sites in breast cancer cells; Lower RIPK1/-3/MLKL expression found to be linked with reduced breast cancer tumorigenicity due to impairment of NF-κβ-mediated pro-tumor growth cytokines secretion	[142]
		Pancreatic adenocarcinoma (PAC)	Tissue samples from 80 human PAC patients; AsPC1, PANC1, and MIA PaCa-2 pancreatic cancer cell lines; Kras^G12D^ PDEC/ FC1242 cells xenografted C57BL/6 mice	Decreased MLKL expression was strongly correlated with cancer progression with diminished necroptosis, MLKL serves as a therapeutic targets in PAC cells, RIPK1/-3 dependent necroptosis signaling promotes pancreatic tumorigenicity through enhancing CXCL1 and Mincle-induced adaptive immune suppression in tumor-infiltrating myeloid cells; Higher RIPK3 and MLKL levels promotes the pancreatic cancer cells migration and invasion via the activation of CXCL5-CXCR2 axis	[145]

## Data Availability

No new data were created or analyzed in this study. Data sharing is not applicable to this article.

## Abbreviations

3-BrPA	3-Bromopyruvate
5-AC	5-azacytidine
6-OHDA	6-hydroxydopamine
Aβ	Amyloid-β
AC	Adenocarcinomas
AD	Alzheimer’s diseases
ADKFM	Adenine diet kidney fibrosis model
ALD	Alcoholic liver disease
ALS	Amyotrophic lateral sclerosis
AKI	Acute kidney injury
AML	Acute myeloid leukemia
APOE	Apolipoprotein E
ARDS	Acute respiratory distress syndrome
BMDMs	Bone marrow-derived macrophages
CaMKII	Ca^2+^/calmodulin-dependent protein kinase II
Ch25h	Cholesterol 25-hydroxylase
cIAPs	Cellular inhibitor of apoptosis proteins
CKD	Chronic kidney disease
CLL	Chronic lymphocytic leukemia
CNLS	Ceramide nano-liposomes
COPD	Chronic obstructive pulmonary disease
CYLD	Cylindromatosis
CXCL1	C-X-C Motif Chemokine Ligand 1
DAC	5-aza-2’-deoxycytidine
DAI	DNA-dependent activator of IRFs
DAMPs	Damage-associated molecular patterns
DAPE	1,2-Diarachidonoyl-sn-glycero-3-phosphoethanolamine
DMA	Disease-associated microglia
DMF	Dimethyl fumarate
DRs	Death receptors
Drp1	Dynamin-related protein 1
EAE	Experimental allergic encephalomyelitis
FADD	Fas-associated protein with death domain
FA-AKI	Folic acid-induced AKI model
FAO	Fatty acid β-oxidation
FLIPL	FLICE-like inhibitory protein long form
Fn-14	Fibroblast growth factor-inducible 14
FTD	Frontotemporal dementia
Fer-1	Ferrostatin-1
GAT	Ganoderic acid T
GLUD1	Glutamate dehydrogenase 1
GSK	GlaxoSmithKline
HD	Huntington disease
HFFC	High in fat, fructose, and cholesterol
HFD	High-fat diet
HMGB1	High-mobility group box 1
HPβCD	2-hydroxypropyl-β-cyclodextrin
ICBs	Immune-checkpoint blockades
IFN-γ	Interferon-gamma
IPF	Idiopathic pulmonary fibrosis
I/R	Ischemia/reperfusion
MAPKs	Mitogen-activated protein kinases
MCAO	Middle cerebral artery occlusion
MCD	Methionine–choline-deficient
MLKL	Mixed-lineage kinase domain-like
MMS	Methyl methanesulfonate
MS	Multiple sclerosis
LAD	Left anterior descending coronary artery
LDL	Low-density lipoprotein
LOAD	Late-onset AD
LPS	Lipopolysaccharides
LRRK2	Leucine-rich repeat kinase 2
NAFLD	Non-alcoholic fatty liver disease
NASH	Non-alcoholic steatohepatitis
NBC1	Necroptosis-blocking compound 1
Nec-1	Necrostatin-1
NF-κβ	Nuclear factor kappa B
NEMO	NF-κB essential modulator
NOX4	NADPH Oxidase 4
NSA	Necrosulfonamide
NSCLC	Non-small cell lung cancer tissue
PAMPs	Pathogen-associated molecular patterns
PBPs	Protein-bound polysaccharides
PCD	Programmed cell death
PD	Parkinson’s disease
PDA	Pancreatic ductal adenocarcinomas
PGAM5	Phosphorylate phosphoglycerate mutase family member 5
PI3K/AKT	Phosphoinositide-3-kinase–protein kinase B/Akt
Poly I:C	Polyionisic:polycytidylic acid
PYCARD	PYD And CARD Domain Containing
RA	Rheumatoid arthritis
RGMB	Repulsive guidance molecule B
RHIM	RIP homotypic interaction motif
RIPK	Receptor-interacting protein kinase
RNI	Reactive-nitrogen intermediates
SHIP	Src homology 2 domain-containing inositolphosphate 5’-phosphatase
SIRS	Sepsis/systemic inflammatory associated disorder
SCC	Squamous cell carcinoma
SCI	Spinal cord neuronal injury
TAB	TAK1-binding protein
tGCI	Transient global cerebral ischemia
TNF	Tumor necrosis factor
TLRs	Toll-like receptors
TRADD	TNFR-associated death domain
TRAF	TNFR-associated factor
TWEAK	Tumor necrosis factor-related weak inducer of apoptosis
UUO	Unilateral ureteral obstruction
ZBP1	Z-DNA binding protein 1
Z-VAD-FMK	Carbobenzoxy-valyl-alanyl-aspartyl-[O-methyl]- fluoromethylketone
